# Isobutyrate Confers Resistance to Inflammatory Bowel Disease through Host–Microbiota Interactions in Pigs

**DOI:** 10.34133/research.0673

**Published:** 2025-05-08

**Authors:** Xiuyu Fang, Haiyang Liu, Junling Liu, Yongqing Du, Zihan Chi, Yiqi Bian, Xuan Zhao, Teng Teng, Baoming Shi

**Affiliations:** College of Animal Science and Technology, Northeast Agricultural University, Harbin 150030, People’s Republic of China.

## Abstract

Supplementation with short-chain fatty acids (SCFAs) is a potential therapeutic approach for inflammatory bowel disease (IBD). However, the therapeutic effects and mechanisms of action of isobutyrate in IBD remain unclear. Clinical data indicate that the fecal levels of isobutyrate are markedly lower in patients with Crohn’s disease than in healthy controls. Compared with healthy mice and healthy pigs, mice and pigs with colitis presented significantly lower isobutyrate levels. Furthermore, the level of isobutyrate in pigs was significantly negatively correlated with the disease activity index. We speculate that isobutyrate may play a crucial role in regulating host gut homeostasis. We established a model of dextran sulfate sodium-induced colitis in pigs, which have gastrointestinal structure and function similar to those of humans; we performed multiomic analysis to investigate the therapeutic effects and potential mechanisms of isobutyrate on IBD at both the animal and cellular levels and validated the results. Phenotypically, isobutyrate can significantly alleviate diarrhea, bloody stools, weight loss, and colon shortening caused by colitis in pigs. Mechanistically, isobutyrate can increase the relative abundance of *Lactobacillus reuteri*, thereby increasing the production of indole-3-lactic acid, regulating aryl hydrocarbon receptor expression and downstream signaling pathways, and regulating Foxp3^+^ CD4^+^ T cell recruitment to alleviate colitis. Isobutyrate can directly activate G protein-coupled receptor 109A, promote the expression of Claudin-1, and improve intestinal barrier function. In addition, isobutyrate can increase the production of intestinal SCFAs and 3-hydroxybutyric acid and inhibit the TLR4/MyD88/NF-κB signaling pathway to suppress intestinal inflammation. In conclusion, our findings demonstrate that isobutyrate confers resistance to IBD through host–microbiota interactions, providing a theoretical basis for the use of isobutyrate in alleviating colitis.

## Introduction

Inflammatory bowel disease (IBD) is a special type of chronic inflammatory disease of the intestines that mainly includes Crohn’s disease (CD) and ulcerative colitis (UC) [1]. Patients with IBD are prone to recurrent abdominal pain and bloody diarrhea, often accompanied by decreased levels of short-chain fatty acids (SCFAs) in the intestines and dysbiosis of the intestinal microbiota [2,3]. IBD may cause both physical and emotional harm to patients and significantly decrease quality of life, and expensive long-term treatment is needed to alleviate symptoms [4,5]. Although various medications, such as corticosteroids, immunosuppressants, and antibiotics, are available for treating IBD, prolonged usage of these medications frequently results in adverse effects such as nausea, abdominal pain, and fever [6,7]. Therefore, studies with a focus on finding healthier and safer treatment strategies are needed.

SCFAs play a crucial role in regulating the health of the host [8–11]. However, unlike SCFAs (acetate, propionate, and butyrate) derived from fermentable dietary fibers, branched SCFAs (BSCFAs) (isobutyrate, isovalerate, etc.) are produced through protein hydrolytic metabolism, are present at relatively low levels in the body, are difficult to detect, and are not widely studied by researchers [12]. A few studies have shown that isobutyrate can regulate lipid metabolism in the liver and may indirectly contribute to the alleviation of atherosclerosis, whereas a high-protein diet may improve intestinal health by promoting the production of ketones and BSCFAs [13–16]. Analysis of publicly available clinical data revealed that isobutyrate levels in the feces of patients with CD are lower than those in the feces of healthy controls [17]. Studies have shown that UC, irritable bowel syndrome, colorectal cancer, and other intestinal diseases are accompanied by decreases in the levels of isobutyrate [18–20]. After treatment, the isobutyrate levels in patients significantly increase [21]. In addition, in the dextran sulfate sodium (DSS)-induced colitis mouse model we constructed, the level of isobutyrate was decreased compared to that in control mice. On the basis of the above research, we speculate that supplementation with isobutyrate, the levels of which are significantly reduced in IBD patients, may be an effective approach to alleviate IBD.

SCFAs are important mediators of gut microbiota–host interactions, and they originate from the gut microbiota and modulate gut microbial homeostasis [22,23]. SCFA levels in the feces of patients with IBD are reduced, and the administration of butyrate can alter disease status [24,25]. Another clinical study revealed that the abundance of *Lactobacillus* in the gut microbiota is significantly reduced in patients with UC compared to controls and that probiotic supplementation can ameliorate gut dysbiosis and increase the levels of SCFAs such as isobutyrate [26,27]. Other studies have shown that oral propionate can reshape the gut microbiota of mice and thereby improve gut barrier function and vascular calcification [28]. Supplementation with acetate or butyrate can alter the intestinal environment of mice and alleviate damage to the colonic barrier caused by a high-copper diet [29]. SCFAs can also directly regulate G protein-coupled receptors (GPCRs) and activate regulatory T cells, thereby promoting intestinal mucosal immunity and alleviating colitis [30]. Although evidence supports the beneficial effects of SCFAs in maintaining host health, the relationship between SCFAs and IBD is complex and involves intricate interactions among the gut microbiota, intestinal barrier integrity, and gut immunity [31–33]. Therefore, a DSS-induced pig colitis model was established to investigate the anticolitic effect of isobutyrate, and methods such as 16S rRNA sequencing, metagenomics, metabolomics, and small interfering RNA (siRNA) transfection were used to explore the beneficial effects and potential molecular mechanisms of isobutyrate at the animal and cellular levels.

The experimental results indicated that isobutyrate can reshape the intestinal microbiota, increase the relative abundance of probiotics, especially *Lactobacillus reuteri*, thereby promoting the production of indole-3-lactic acid (ILA), regulating aryl hydrocarbon receptor (AhR) and downstream signaling pathways, inducing the generation of Foxp3^+^ CD4^+^ T cells, and improving the intestinal barrier and immune function. Considering that isobutyrate is a well-studied isomer of the anti-inflammatory SCFA butyrate [34], we speculate that isobutyrate may regulate intestinal barrier and immune function through the GPCR pathway and the TLR4/MyD88/NF-κB pathway, and our experiments confirmed these speculations. Notably, the gastrointestinal system of pigs is more similar to that of humans [35,36], and findings with this rarely reported pig DSS-induced colitis model further support the clinical efficacy of isobutyrate in the treatment of IBD and lay a scientific foundation for future exploration of the interaction between isobutyrate and the gut microbiota in the treatment of colitis.

## Results

### Isobutyrate levels are significantly decreased in CD patients and DSS-induced colitis of mice and pigs

**Fig. 1.**
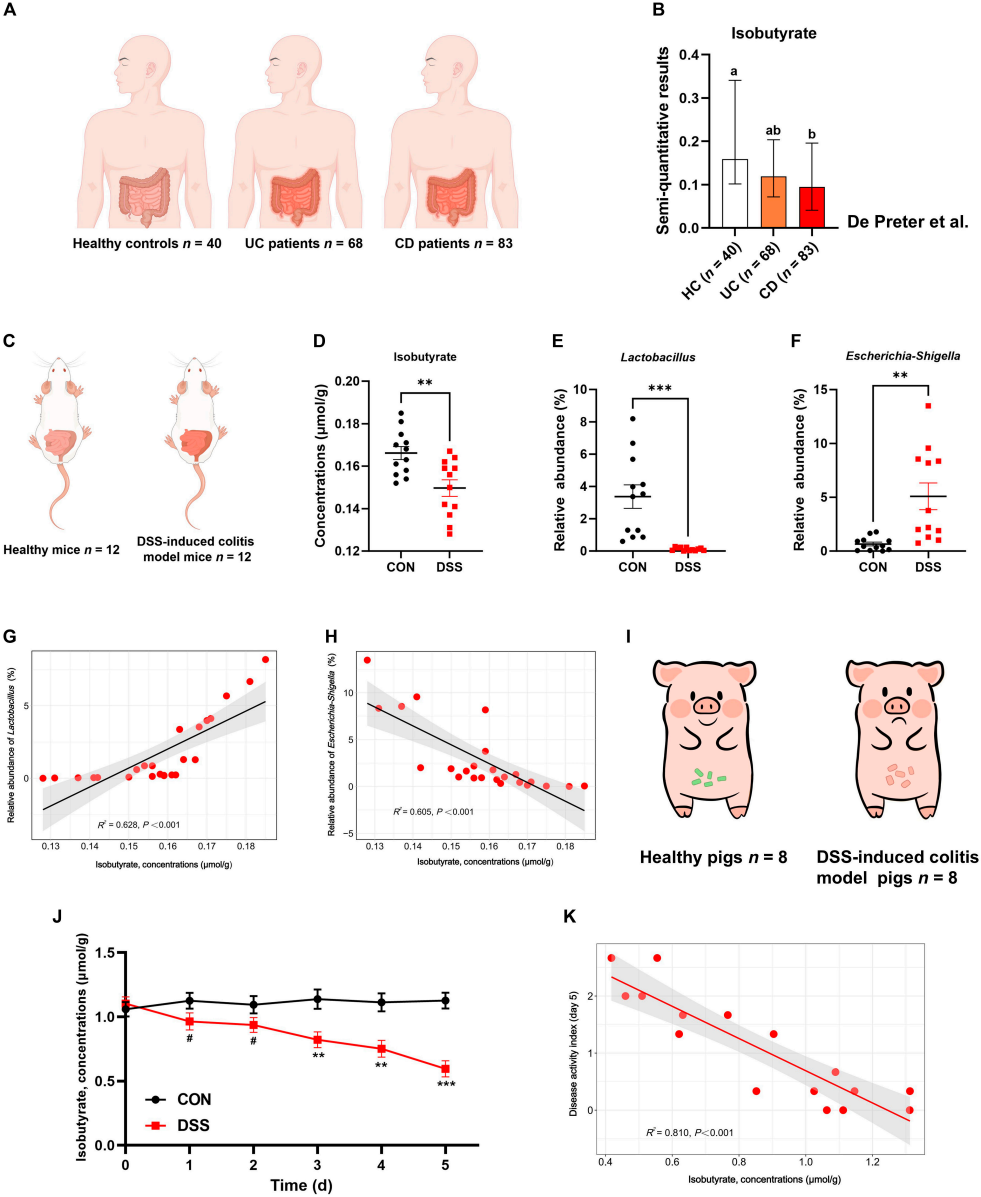
Isobutyrate levels are significantly decreased in CD patients and DSS-induced colitis of mice and pigs. (A and B) Levels of isobutyrate in the feces of patients with IBD. The clinical data originate from De Preter et al. [17] The data are presented as the means ± min/max. (C and D) Colonic isobutyrate levels in DSS-induced colitis of mice (*n* = 12). (E and F) The relative abundances of *Lactobacillus* and *Escherichia-Shigella* in the mouse colon (*n* = 12). (G and H) Correlations between isobutyrate concentration and *Lactobacillus* or *Escherichia-Shigella* abundance in mice (*n* = 12). (I and J) Fecal isobutyrate levels in pigs with DSS-induced colitis (*n* = 8). (K) Correlation between isobutyrate concentration and DAI in pigs (*n* = 8).

Public clinical data analysis revealed that isobutyrate levels are decreased to a certain extent in patients with CD compared with healthy controls (Fig. 1A and B). Therefore, we established a DSS-induced colitis mouse model and measured the levels of isobutyrate in the colonic digesta of the mice (Fig. 1C and Fig. S1). The results revealed a significant decrease in isobutyrate levels in the mice with colitis (Fig. 1D; *P* < 0.05). Further analysis of the intestinal microbiota in the mice revealed a significant decrease in the abundance of *Lactobacillus* and *Escherichia-Shigella* in the mice with colitis (Fig. 1E and F; *P* < 0.05). There was a significant positive correlation between isobutyrate levels and the relative abundance of *Lactobacillus* in the mice with colitis (Fig. 1G; *P* < 0.05). Similarly, the level of isobutyrate was significantly negatively correlated with the relative abundance of *Escherichia-Shigella* (Fig. 1H; *P* < 0.05). In our study, we monitored the dynamic changes in isobutyrate levels in healthy pigs and pigs with DSS-induced colitis (Fig. 1I). The results revealed that as colitis progressed, the levels of isobutyrate in pig feces gradually decreased, with a decrease in the first 2 days (0.05 ≤ *P* < 0.1) and a significant decrease from day 3 to day 5 (Fig. 1J; *P* < 0.05). Analysis of isobutyrate levels and the disease activity index (DAI) in colitis model pigs revealed a significant negative correlation between isobutyrate levels and the DAI (Fig. 1K; *P* < 0.05).

### Prefeeding with isobutyrate significantly alleviates DSS-induced colitis in pigs

**Fig. 2.**
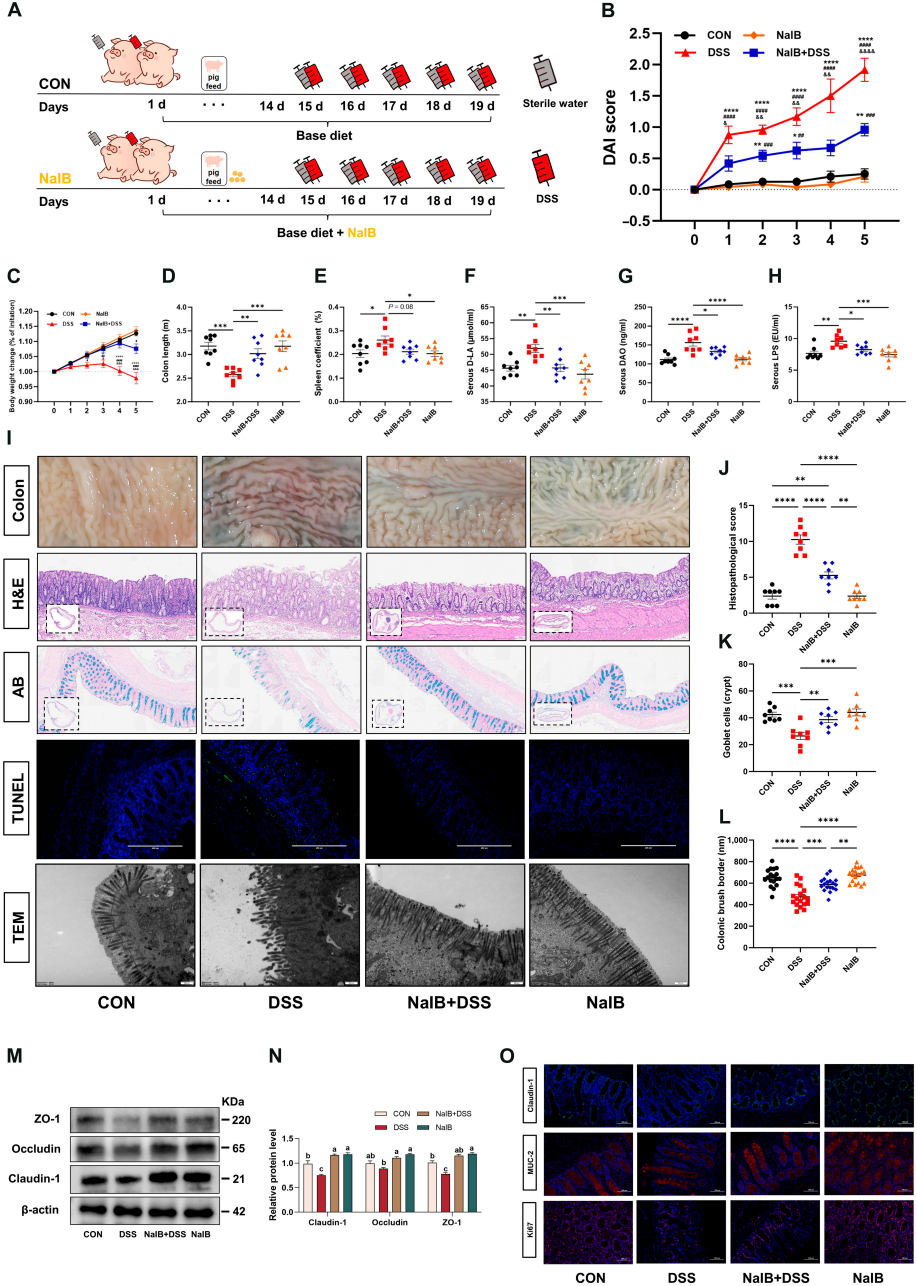
Prefeeding with isobutyrate significantly alleviates DSS-induced colitis in pigs. (A) For 14 days before the experiment, the CON group consumed a basic diet, whereas the NaIB group received a basic diet supplemented with 1,000 mg/kg sodium isobutyrate. On the 15th day, DSS-induced colitis model generation was performed, with 8 pigs randomly selected from the CON/NaIB group for DSS challenge (4% DSS in 100 ml of sterile water) via gavage, while the remaining 8 were gavaged with an equal amount of sterile water. The DSS challenge lasted for 5 days, with a double dose administered on the first day. (B) Plot of the DAI values of pigs after DSS treatment against the number of days (*n* = 8). (C) Body weight change of pigs after DSS treatment in the CON, DSS, NaIB + DSS, and NaIB groups (^*^P < 0.05 vs. CON group; ^#^*P* < 0.05 vs. NaIB group; ^&^*P* < 0.05 vs. NaIB + DSS group). (D and E) Comparison of colon length and the spleen coefficient in pigs after DSS treatment (*n* = 8). (F to H) Measurement of D-LA, DAO, and LPS in pig serum (*n* = 8). (I) Representative images of the pig mucosa, H&E-stained, Alcian blue-stained inner mucus layers, TUNEL, and TEM images of the colon (*n* = 6 to 8). (J and K) Histopathological scoring and goblet cell counts of the colon. (L) Measurement of the colonic brush border (*n* = 6). (M and N) Western blot of TJ proteins in the pig colon (*n* = 3). (O) Representative images of colonic immunofluorescence staining with Claudin-1, MUC-2, and Ki67 antibodies (*n* = 6).

On the basis of these results, we established a DSS-induced colitis model in pigs to investigate the role of isobutyrate in IBD (Fig. 2A). The results revealed that during the DSS challenge period, the DAI of the DSS group began to increase significantly from the first day (*P* < 0.05) and significantly differed from those of the other 3 groups (Fig. 2B and Table S1). The DAI of the sodium isobutyrate (NaIB) + DSS group significantly differed from that of the CON group on the second day of challenge (*P* < 0.05). Pig body weights changed significantly between the DSS and CON groups on the second day of treatment (Fig. 2C). Starting on the third day, the body weight change of the DSS group significantly differed from that of the other 3 groups (*P* < 0.05). In contrast, the body weight change of the NaIB + DSS group significantly differed from that of the CON group only on the fifth day. The diarrhea score, weight score, and hematochezia score results are detailed in Table S1. After the pigs were slaughtered, the length of the colon of each pig was measured. The DSS group had a significantly shorter colon length (*P* < 0.05), whereas the colon length of the NaIB + DSS group did not differ significantly from that of the other 2 groups. (Fig. 2D). The DSS group pigs had enlarged spleens, whereas the spleen size of the NaIB + DSS pigs and CON pigs were not significantly different (Fig. 2E). Furthermore, we observed the colonic mucosal morphology of pigs and found that the colonic mucosa of pigs in the DSS group was severely damaged, with ulcers and redness, whereas the colonic mucosa of the NaIB + DSS group only showed mild redness (Fig. 2I). Further observation of the colon with H&E staining and Alcian blue staining revealed that in the DSS group, crypt loss, goblet cell destruction, reduced mucus, inflammatory cell infiltration, and high histological damage scores were observed (Fig. 2I to K). Additionally, H&E staining across different regions of the colon revealed varying degrees of damage throughout the entire colonic segment (Figs. S2 and S3). Compared with the DSS group, the NaIB + DSS group presented significant alleviation of symptoms, with less mucus reduction and more goblet cells. Moreover, we analyzed the colonic mucosa via terminal deoxynucleotidyl transferase-mediated dUTP nick end labeling (TUNEL) assays and transmission electron microscopy (TEM). The TUNEL results revealed that the DSS group exhibited a high degree of colonic cell apoptosis, whereas the NaIB + DSS group exhibited a lower degree of apoptosis (Fig. 2I). TEM revealed that the colonic brush border in the DSS group was disordered and scattered, with shrinking microvilli and unclear tight junctions (TJs) (Fig. 2L). In contrast, although there was some degree of shrinkage in the NaIB + DSS group, the difference between the NaIB + DSS group and the CON group was not significant, and TJs were clearly visible in the NaIB + DSS group. Compared with those in the NaIB + DSS group, the colonic brush border in the DSS group was significantly shorter (*P* < 0.05). The levels of D-lactic acid (D-LA), diamine oxidase (DAO), and lipopolysaccharide (LPS) in the serum of the DSS group were significantly greater than those in the CON group (*P* < 0.05; Fig. 2F to H). However, there was no significant difference between the NaIB + DSS group and the CON group. Furthermore, we assessed the levels of colonic TJ proteins and found that, compared with those in the CON group, the levels of Claudin-1, Occludin, and ZO-1 in the DSS group were significantly lower (*P* < 0.05; Fig. 2M and N). The representative immunofluorescence images indicated that compared with those in the CON group, the fluorescence intensity and staining area of both Ki67 and MUC2 were reduced in the DSS group. Conversely, the NaIB + DSS group exhibited no significant difference from the CON group in terms of fluorescence intensity or staining area (Fig. 2O).

### Prefeeding with isobutyrate significantly increases the relative abundance of probiotics, especially *L. reuteri*, while promoting the production of SCFAs and beneficial metabolites

**Fig. 3.**
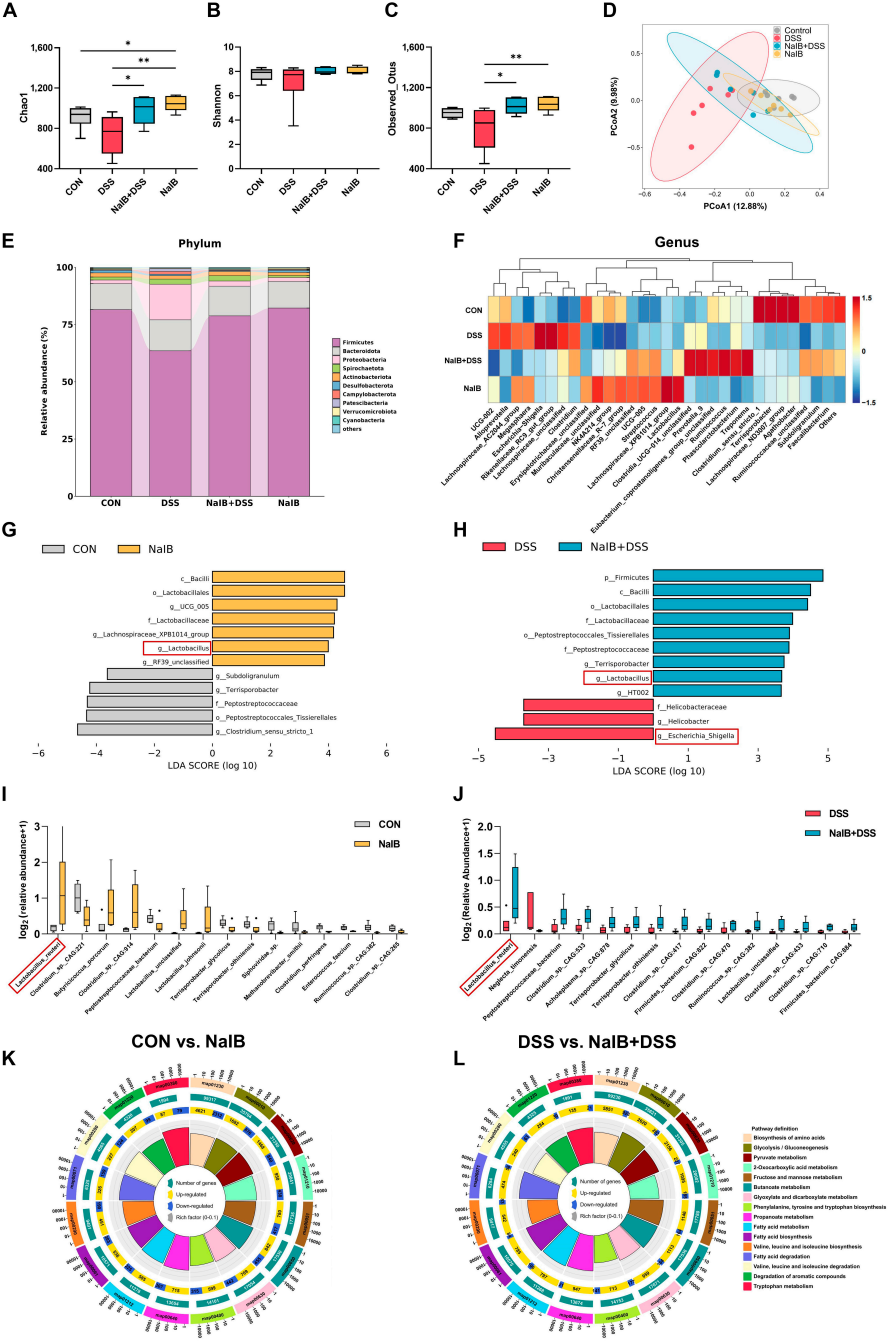
Effects of isobutyrate on the intestinal microbiota of pigs with DSS-induced colitis. (A to C) Comparison of the Chao1, Shannon, and Observed_Otus indices of the gut microbiota in the CON, DSS, NaIB + DSS, and NaIB groups (the same below). (D) Pig microbial composition PCoA plot. (E) Pig colonic microbiota phylum and genus abundances. (F) Relative abundance of the colonic microbiota at the genus level in pigs. (G and H) Microbial abundance differences were analyzed via linear discriminant analysis effect size (LEfSe). (I and J) Relative abundance of the top 15 species with significant differences. (K and L) KEGG pathway enrichment analysis of the CON, DSS, NaIB + DSS, and NaIB groups. *n* = 6.

The gut microbiota and its metabolites influence intestinal barrier function. Further detection of the colonic digesta via 16S rRNA analyses revealed that the α diversity index (Chao1, Shannon, and Observed_Otus) in the DSS group was significantly lower than that in the CON group, whereas there was no significant difference between the NaIB + DSS group and the CON group (*P* < 0.05; Fig. 3A to C). The principal coordinate analysis (PCoA) results indicated that the microbiota in the DSS group significantly differed from those in the other 3 groups (Fig. 3D). Further analysis revealed an increase in the abundance of Proteobacteria and a decrease in the abundance of Firmicutes at the phylum level in the DSS group (Fig. 3E). At the genus level, *Lactobacillus* was present at a significantly greater abundance in the NaIB + DSS group than in the DSS group, and the abundance of *Escherichia-Shigella* in the DSS group was significantly greater (*P* < 0.05; Fig. 3F to H). A comparison between the NaIB group and the NaIB + DSS group revealed a similar significant trend in the change in *Lactobacillus* abundance. Furthermore, metagenomic analysis was used to assess differences in the gut microbiota among the CON, DSS, NaIB + DSS, and NaIB groups (Fig. S4). The top 15 species with significant differences in abundance were selected, and their abundance in the different groups is displayed in box plots (*P* < 0.05; Fig. 3I and J). The relative abundance of *L. reuteri* was significantly decreased in the DSS group (*P* < 0.05; Fig. S5A), while prefeeding with isobutyrate led to a significant increase in the relative abundance of *L. reuteri* in both the NaIB and NaIB + DSS groups. Furthermore, *L. reuteri* had the highest relative abundance among all the significantly altered bacterial species. At the same time, we used the random forest model to analyze the top 100 core strains ranked by relative abundance. We found that the biomarker score of *L. reuteri* ranked second, and its relative abundance across groups was consistent with changes in host health status (Fig. S5B). In addition, we analyzed the functional profile of the strains through the Kyoto Encyclopedia of Genes and Genomes (KEGG) pathway and found that after the addition of isobutyrate, the microbial communities with differential abundance were significantly enriched in pathways such as tryptophan metabolism; fatty acid metabolism; valine, leucine, and isoleucine degradation, etc. (Fig. 3K and L).

**Fig. 4.**
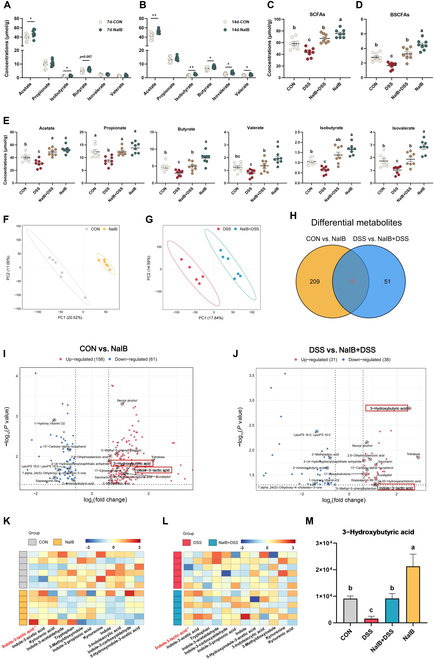
Effects of prefeeding with isobutyrate on the metabolome of pigs with DSS-induced colitis. (A) SCFA content in fresh feces of pigs on the 7th day. (B) SCFA content in fresh feces of pigs on the 14th day. (C to E) Effects of isobutyrate prefeeding on SCFA levels in pigs with DSS-induced colitis. (F and G) PLS-DA of metabolite compositions in the CON, DSS, NaIB + DSS, and NaIB groups (the same below). (H) Differentially abundant metabolites in the normal state and DSS-induced colitis state after isobutyrate prefeeding. (I) Colonic metabolites in the CON and NaIB groups. (J) Colonic metabolites in the DSS and NaIB + DSS groups. (K and L) Heatmap of key metabolites of tryptophan. (M) Content of the colonic metabolite 3-HB. *n* = 6.

We examined the SCFAs and small-molecule metabolites in the colonic contents to identify key metabolic products of the functional microbiota. After pretreatment with isobutyrate, we separately analyzed the SCFAs in fresh feces that were collected on days 7 and 14. The results revealed that the isobutyrate levels were significantly increased in the NaIB group on day 7 (*P* < 0.05; Fig. 4A), with a significant increasing trend in acetate and butyrate (0.05 < *P* < 0.01). On day 14, the acetate, isobutyrate, butyrate, isovalerate, and valerate levels were significantly different (*P* < 0.05; Fig. 4B). After DSS-induced colitis was established in the pigs, we analyzed the colonic digesta and found that the levels of most SCFAs were significantly lower in the DSS group than in the CON group (*P* < 0.05; Fig. 4C to E). In contrast, the effects in the NaIB + DSS group were significantly alleviated compared with those in the DSS group (*P* < 0.05). Additionally, the addition of isobutyrate under normal conditions led to a significant increase in SCFA levels across the gut (*P* < 0.05). Nontargeted metabolomics revealed that prefeeding with isobutyrate led to significant differences in the gut microbiota metabolite levels under both normal and DSS-induced colitis conditions (*P* < 0.05; Fig. 4F and G). In the CON group and the NaIB group, there were 227 significantly differentially abundant metabolites, whereas in the DSS group and the NaIB + DSS group, there were 69 significantly differentially abundant metabolites (Fig. 4H). To more stringently select differentially abundant small-molecule metabolites, we sought those that exhibited consistent changes across the 2 groups. By integrating the variable importance in the projection (VIP) values and fold change data, we discovered that ILA and 3-hydroxybutyric acid (3-HB) levels were significantly altered by the addition of isobutyrate (*P* < 0.05; Fig. 4I, J, and M). There was a marked increase in *Lactobacillus* and *L. reuteri* abundance in the colon. We focused particularly on tryptophan metabolites, and the results indicated that only ILA exhibited significant changes (*P* < 0.05; Fig. 4K and L).

**Fig. 5.**
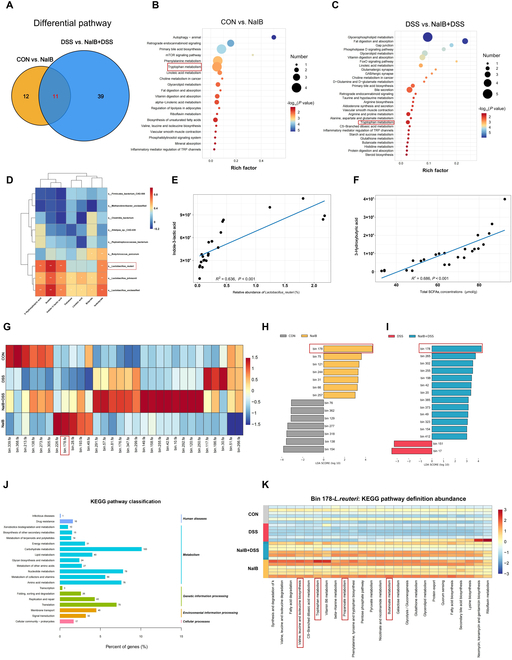
Metabolite functional analysis and metagenome binning analysis of the colonic digesta. (A) Differential KEGG pathways in the normal state and DSS-induced colitis state after isobutyrate prefeeding. (B) KEGG pathway enrichment analysis of the CON and NaIB groups. (C) KEGG pathway enrichment analysis of the DSS and NaIB + DSS groups. (D) Correlations between significantly differentially abundant metabolites and differentially abundant bacterial species. (E) ILA concentration and its association with the relative abundance of *L. reuteri*. (F) 3-HB concentration and its association with SCFAs. (G) Top 30 bins by relative abundance. (H and I) LEfSe was used to analyze the differences in bin abundance. (J and K) KEGG pathway classification and differential abundance analysis of *L. reuteri. n* = 6.

Further analysis of metabolite-related KEGG pathways revealed that tryptophan metabolism, linoleic acid metabolism, biosynthesis of amino acids, bile secretion, and protein digestion and absorption, among others, were significantly enriched under both normal and colitis conditions (Fig. 5A to C). In addition, we conducted a correlation analysis and found that SCFA levels and 3-HB levels were significantly positively correlated (*P* < 0.05; Fig. 5D). Analysis of the correlation between the abundance of microbes in the gut and the levels of small-molecule metabolites revealed that isobutyrate levels in the colon were significantly positively correlated with the abundance of *Lactobacillu*s and *Butyricicoccus*, whereas 3-HB and ILA levels were significantly positively correlated with *Lactobacillus* abundance (*P* < 0.05). Similarly, a strong positive correlation was observed between *L. reuteri* abundance and the levels of both ILA and 3-HB (*P* < 0.05; Fig. 5E and F). Furthermore, we performed metagenomic binning analysis to explore the relative abundance and functional enrichment of a single strain. We conducted statistical analysis on the top 30 bins in terms of abundance and performed linear discriminant analysis effect size (LEfSe) analysis on the 2 groups separately (Fig. 5G). The results revealed that bin178 presented the most significant differential changes after the addition of isobutyrate (Fig. 5H and I). By identifying this bin as *L. reuteri*, its status as the core strain with the most significant abundance changes was confirmed. Furthermore, we classified KEGG functions and conducted a relative quantitative analysis of KEGG pathways enriched in *L. reuteri* (Fig. 5J and K). The results revealed that after the addition of isobutyrate, *L. reuteri* in the NaIB + DSS and NaIB groups presented relatively high enrichment of pathways such as tryptophan metabolism; butanoate metabolism; valine, leucine, and isoleucine biosynthesis; and propanoate metabolism. The addition of isobutyrate promoted the tryptophan metabolism pathway in *L. reuteri*, which is consistent with the metagenomic and metabolomic results at the host microbiota level. In conclusion, we found that isobutyrate can promote the enrichment of *L. reuteri* and thereby promote the production of ILA. Therefore, we further investigated the downstream signaling pathways regulated by this gene.

### Prefeeding isobutyrate activates AhR and its downstream signaling pathway and drives of Foxp3^+^ CD4^+^ T cells to alleviate colitis

**Fig. 6.**
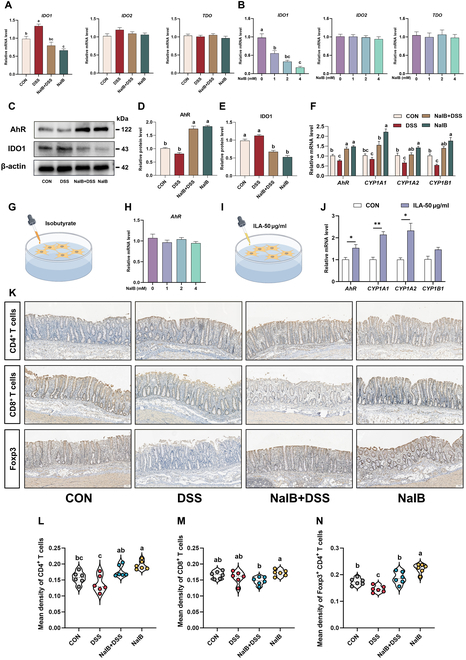
Prefeeding isobutyrate activates AhR and its downstream signaling pathway and drives of Foxp3^+^ CD4^+^ T cells to alleviate colitis. (A) Pig colon mRNA expression of *IDO1*, *IDO2*, and *TDO* (*n* = 8). (B) IPEC-J2 cell mRNA expression of *IDO1*, *IDO2*, and *TDO* (*n* = 3). (C to E) Western blot of AhR and IDO1 in the pig colon (*n* = 3). (F) AhR pathway-related mRNA expression in the pig colon (*n* = 8). (G and H) Effects of isobutyrate on the relative expression of *AhR* in IPEC-J2 cells (*n* = 3). (I and J) Treatment with 50 μg/ml ILA activated the AhR signaling pathway in IPEC-J2 cells (*n* = 3). (K) Representative immunohistochemistry images of colonic sections stained with antibodies against CD4^+^ T cells, CD8^+^ T cells, and Foxp3^+^ CD4^+^ T cells (*n* = 6). (L to N) Immunohistochemical analysis of immune cells (*n* = 6). The results are expressed as the average optical density (AOD).

Although multiomic results indicate that isobutyrate promotes the growth of *L. reuteri* and facilitates its production of ILA, the mechanism of action is not clear. Our in vitro experiments confirmed that the cocultivation of isobutyrate and *L. reuteri* does not promote its growth or the production of ILA (Fig. S6), which means that the increase in the abundance of *L. reuteri* in vivo induced by isobutyrate requires a complex intestinal microenvironment. In addition, adding extra tryptophan to the medium can promote the growth of *L. reuteri* and enhance the production of ILA. Previous studies have shown that limiting the consumption of tryptophan by intestinal epithelial cells may promote the proliferation of *Lactobacillus* spp. [37]. Indoleamine 2,3-dioxygenase 1 (IDO1), IDO2, and tryptophan-2,3-dioxygenase (TDO) are key rate-limiting enzymes for tryptophan catabolism. Therefore, we assayed them in pigs and porcine intestinal epithelial (IPEC-J2) cells. Prior to this, we determined the maximum safe dose of isobutyrate in IPEC-J2 cells to be 4 mM (Fig. S7). The results showed that isobutyrate significantly inhibited the activity of IDO1, which may be important for its ability to promote *L. reuteri* growth (*P* < 0.05; Fig. 6A and B). ILA is an agonist of the AhR signaling pathway; for this reason, we examined changes in this signaling pathway. Compared with those in the DSS group, the AhR protein levels in the NaIB + DSS group were significantly greater, whereas IDO1 protein levels were significantly decreased (*P* < 0.05; Fig. 6C to E). Furthermore, we examined the mRNA expression levels of genes downstream of AhR (*CYP1A1*, *CYP1A2*, and *CYP1B1*) in the colon. The results revealed that the NaIB + DSS group presented significantly upregulated expression of the *CYP1A1*, *CYP1A2*, and *CYP1B1* genes (*P* < 0.05; Fig. 6F), further indicating that AhR was activated in colonic tissues. However, we were unable to determine whether isobutyrate activates the AhR receptor directly or indirectly by promoting the growth of *L. reuteri* for ILA production. We therefore used IPEC-J2 cells for our tests and showed that isobutyrate does not directly activate the AhR receptor under in vitro conditions (*P >* 0.05; Fig. 6G and H). In contrast, under in vitro conditions, ILA activated AhR receptors and promoted the expression of the downstream genes *CYP1A1* and *CYP1A2* (Fig. 6I and J). Studies have shown that ILA regulates the proliferation of immune cells [38,39]. Therefore, we examined CD4^+^ T cells and CD8^+^ T cells and found that, in the NaIB + DSS and NaIB groups, the number of CD4^+^ T cells significantly increased (*P* < 0.05; Fig. 6K to M). We further investigated Foxp3^+^ CD4^+^ T cells and observed a significant decrease in the DSS group, while its expression was significantly increased in the NaIB + DSS and NaIB groups (*P* < 0.05; Fig. 6N and Fig. S8).

### Effect of isobutyrate prefeeding on GPCRs and the TLR4/MyD88/NF-κB signaling pathway in pigs with DSS-induced colitis

**Fig. 7.**
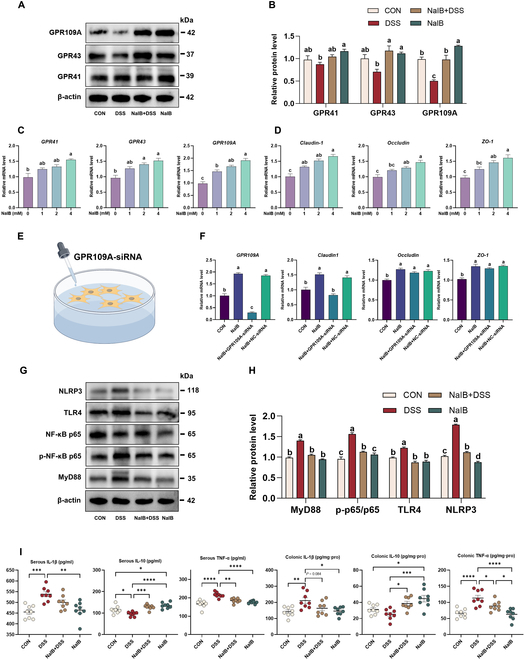
Effects of isobutyrate prefeeding on GPCRs and the TLR4/MyD88/NF-κB signaling pathway in pigs with DSS-induced colitis. (A and B) Western blot of G protein-coupled receptors (GPR41, GPR43, and GPR109A) in the pig colon (*n* = 3). (C and D) Effects of isobutyrate on the relative expression of GPCRs and TJ proteins in IPEC-J2 cells (*n* = 3). (E and F) Effects of isobutyrate on intestinal barrier function after silencing the GPR109A gene (*n* = 3). (G and H) Western blot of TLR4, NLRP3, MyD88, NF-κB, and P-NF-κB in the pig colon (*n* = 3). (I) Concentrations of 3 representative cytokines, IL-1β, IL-10, and TNF-α, in the serum and colon (*n* = 8).

**Fig. 8.**
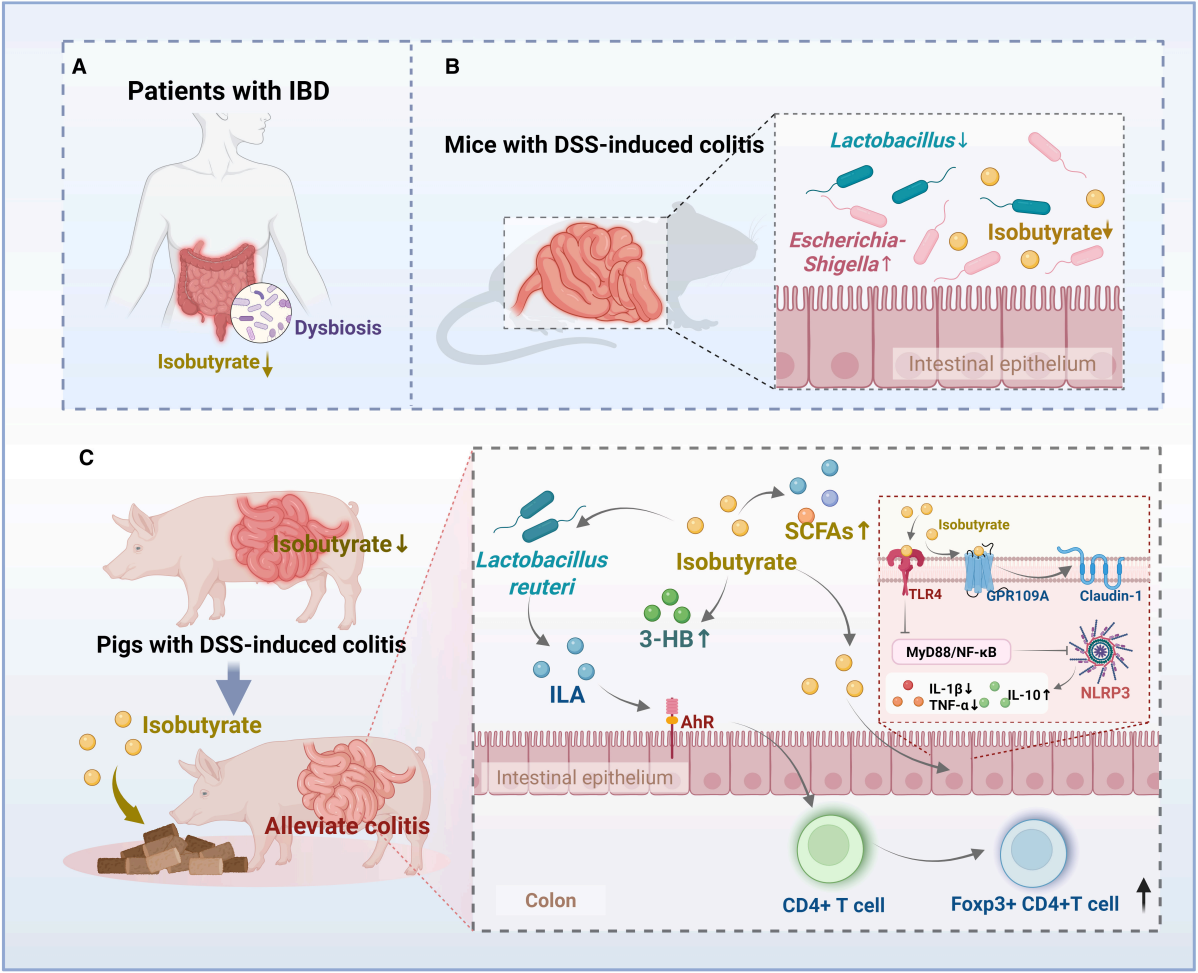
Isobutyrate confers resistance to inflammatory bowel disease through host–microbiota interactions in pigs. Isobutyrate levels are significantly decreased in CD patients (A) and DSS-induced colitis of mice and pigs (B and C), and supplementation with isobutyrate alleviates colitis in piglets. Mechanistically, isobutyrate can increase the relative abundance of *L. reuteri*, thereby increasing the production of indole-3-lactic acid, regulating aryl hydrocarbon receptor expression and downstream signaling pathways, and regulating Foxp3^+^ CD4^+^ T-cell recruitment to alleviate colitis. Isobutyrate can directly activate G protein-coupled receptor 109A, promote the expression of Claudin-1, and improve intestinal barrier function. In addition, isobutyrate can increase the production of intestinal SCFAs and 3-hydroxybutyric acid and inhibit the TLR4/MyD88/NF-κB signaling pathway to suppress intestinal inflammation. In conclusion, our findings demonstrate that isobutyrate confers resistance to IBD through host–microbiota interactions, providing a theoretical basis for the use of isobutyrate in alleviating colitis (created with Biorender.com).

Considering that isobutyrate is a well-studied anti-inflammatory SCFA butyrate isomer, we examined signaling pathway GPCRs. We investigated 3 key GPCRs (GPR41, GPR43, and GPR109A) and found that isobutyrate activated these receptors (*P* < 0.05; Fig. 7A and B), particularly GPR109A. Since isobutyrate modulates the production of SCFAs and 3-HB by the intestinal microbiota, of which butyrate and 3-HB are known to activate GPR109A, we further validated the direct activation of GPCRs by isobutyrate in IPEC-J2 cells. We found that isobutyrate directly activated GPCRs, especially GPR109A (*P* < 0.05; Fig. 7C and D). Furthermore, we found that isobutyrate can affect IPEC-J2 TJ protein expression, which is consistent with the results in pigs. Studies have shown that SCFAs can modulate intestinal barrier function by regulating GPCRs. We silenced the GPR109A gene via the use of GPR109A-siRNA and again examined the expression of intestinal TJ proteins (Fig. 7E). This finding revealed that isobutyrate cannot activate Claudin-1 (Fig. 7F). The TLR4/MyD88/NF-κB pathway is also an important signaling pathway that regulates IBD. The results revealed that isobutyrate inhibited the protein expression of TLR4/MyD88/NF-κB and suppressed the protein expression of the NOD-like receptor thermal protein domain associated protein 3 (NLRP3) inflammasome (*P* < 0.05; Fig. 7G and H). Furthermore, serum and colonic mucosal inflammatory factor levels revealed that isobutyrate suppressed the expression of the proinflammatory factor tumor necrosis factor-α (TNF-α) in both the serum and colonic mucosa but promoted the expression of the anti-inflammatory factor interleukin-10 (IL-10; *P* < 0.05; Fig. 7I). Overall, the molecular mechanism by which isobutyrate alleviates DSS-induced colitis in pigs is shown in Fig. 8.

## Discussion

SCFAs serve as a bridge between the gut microbiota and the host [40,41], exerting positive effects on conditions such as IBD, cardiovascular diseases, and immune system disorders [42,43]. Most studies have shown that colitis is often accompanied by a significant reduction in isobutyrate levels. For example, DSS concentrations ranging from 3% to 5% have been reported to cause a marked decrease in isobutyrate levels [26,44,45]. According to the results of clinical studies and our analysis of colitis model mice, isobutyrate levels are significantly reduced in the colitis state. However, some studies have not found significant changes in isobutyrate levels under certain experimental conditions [46]. These discrepancies may be attributed to differences in DSS concentration, exposure duration, and severity of induced colitis. Higher DSS concentrations, which lead to more severe colitis, may significantly suppress isobutyrate production and increase isobutyrate consumption. To further explore the role of isobutyrate in colitis and its potential therapeutic benefits, we used a relatively high DSS concentration (4%) to induce acute colitis in a pig model. We dynamically monitored changes in isobutyrate levels during the progression of colitis. The results revealed a continuous decrease in isobutyrate levels as the degree of colitis increased. In conclusion, we hypothesize that isobutyrate can improve host intestinal homeostasis and alleviate colitis. Therefore, we conducted experiments for further validation. In the DSS-induced colitis model, the DSS group presented an increased DAI during treatment, which manifested as a decrease in body weight and exacerbated diarrhea and the occurrence of colonic bleeding, which are characteristics that are consistent with IBD; these results confirmed the successful establishment of the inflammatory model [22]. After prefeeding with isobutyrate, the DAI in the NaIB + DSS group was significantly reduced; the DAI increased more gradually and exhibited smaller numerical changes. Symptoms of diarrhea, weight loss, and colonic bleeding began to appear only on the fourth and fifth days. Additionally, isobutyrate alleviated the shortening of the intestines of pigs and the decrease in intestinal density induced by DSS. These findings are key indicators of the efficacy of sodium isobutyrate in alleviating host colitis. The spleen is a crucial immune organ in the body, and the splenic index (the ratio of spleen weight to body weight) has traditionally been a significant indicator for assessing immune system development [47,48]. When inflammation occurs in the body, the spleen may become enlarged, indicating the presence of a disease [49,50]. This could be due to compensatory growth triggered by internal immune stimulation. Prefeeding with isobutyrate may alleviate the excessive burden on immune organs caused by colitis, whereas the DSS group may be in a state of “overload operation”. The colon is an organ that is rich in lymphocytes and is considered one of the most important immune organs [51]. The proportion and length of the colon in the body are important immune phenotypes. Similar to the results for the spleen, the colon weight of the NaIB + DSS group remained relatively stable, whereas the DSS group presented significant swelling and shortening of the colon. These findings demonstrate the protective and anti-inflammatory properties of isobutyrate in pigs. The mucus layer of the colon serves to protect the intestinal tract, and the brush border of the colon facilitates the digestion and absorption of nutrients [52]. This study further conducted histological observations of inflammatory lesion sites. After DSS treatment, the colon exhibited severe mucosal edema and large ulcer areas. The histopathological scores and DAI clinical scores consistently increased. DSS induced colonic crypt loss, promoted goblet cell destruction, reduced mucus, increased inflammatory cell infiltration, and elevated histopathological scores, affecting the entire colon. Interestingly, although the histopathological scores were significantly elevated throughout the entire colon in the DSS group, the scores in the proximal and mid-colon were slightly lower than those in the distal colon. This may be related to the fact that UC primarily manifests in the distal colon [53,54]. Prefeeding with isobutyrate significantly alleviated these symptoms.

IBD is often accompanied by intestinal barrier damage and intestinal dysbiosis [55]. D-LA and DAO are the most direct indicators of increased intestinal permeability. When the levels of these indicators are increased in serum, dysbiosis of the intestinal microbiota can result in the production of excessive LPS [56]. As the intestinal barrier is destroyed, excessive LPS enters the bloodstream, leading to inflammation in the body. After DSS administration, all 3 factors increased to varying degrees. However, supplementation with isobutyrate significantly attenuated these increases, possibly by decreasing intestinal permeability. TJs, which involve multiple protein networks, are the most important mechanism by which cells are connected [57]. TJs constitute the basic structure of the mucosal barrier in the intestinal tissue of animals and can form barriers with selective permeability between adjacent intestinal epithelial cells [58]. Mucosal barrier function depends on TJ proteins [59]. There is a significant correlation between intestinal barrier dysfunction and IBD incidence [60]. A characteristic feature of IBD is increased intestinal barrier permeability, which allows pathogens and their toxins to enter from the intestinal lumen, leading to the onset of the disease [61]. In the DSS group, the expression of the relevant proteins was significantly reduced. After prefeeding with isobutyrate, the expression of these proteins recovered to a certain extent, indicating that they can regulate the expression of TJ proteins to ameliorate gut barrier function.

The gut microbiota plays a pivotal role in regulating host immunity and nutrient metabolism as well as in maintaining the integrity of the intestinal barrier structure [62,63]. The integrity of the gut barrier is influenced by numerous intrinsic and extrinsic factors, including genetics, diet, antibiotics, and aging [40,64]. The gut microbiota constitutes the biological barrier of the intestine [65,66]. In the intestine, the colon is the most densely populated and active site for microbial activity and metabolism, with microbe numbers exceeding 10^13^ [67,68]. The microbiota in the colon, including symbiotic bacteria, probiotics, and pathogenic bacteria, mainly participates in host immune responses and activities such as reactions and modifications to specific drugs, thus playing an important role in intestinal health [69]. IBD is a disease that is caused by abnormal host–microbiota interactions and involves a combination of dysbiosis of the gut microbiota, abnormal immune responses, and impairment of the intestinal mucosal barrier [70,71]. We analyzed the colonic microbiota and found that isobutyrate not only altered the alpha diversity index of the microbiota in pigs with DSS-induced colitis but also significantly affected the composition of the intestinal microbiota in normal pigs and pigs with DSS-induced colitis. DSS increased the abundance of Proteobacteria and decreased the abundance of Firmicutes, whereas the addition of isobutyrate alleviated these effects. Proteobacteria frequently negatively impact the health of the host, whereas Firmicutes mainly comprises beneficial bacteria that are beneficial to the host. Firmicutes can produce SCFAs, such as acetate and butyrate [72]. *Escherichia-Shigella* is a group of commonly found pathogens whose abundance significantly increases in the pig colon after treatment with DSS. However, isobutyrate supplementation can alleviate this condition and significantly increase the population of probiotic *Lactobacillus*. This microbiota shift aligns with that observed in colitis mice [26]. To better elucidate the core microbes that are involved in host regulation, we performed metagenomic analysis at the species level. We compared the relative abundances of the top 15 species before and after DSS treatment. Our analysis revealed that *L. reuteri* was the most abundant species. Furthermore, our results demonstrated that isobutyrate significantly increased the abundance of *L. reuteri*, regardless of whether the animals were treated with DSS. Moreover, the abundance of *L. unclassified*, *L. johnsonii*, and *Butyricicoccus porcorum* also increased to a certain degree. Thus, we hypothesize that isobutyrate may increase the relative abundance of *Lactobacillus*, particularly *L. reuteri*, which could be a significant factor in ameliorating host colitis. Studies have shown that changes in the gut microbiota lead to changes in intestinal metabolites in IBD patients, especially SCFAs, indole derivatives, and bile acids, which are involved in metabolic pathways that are related to the pathogenesis of IBD [73,74]. This study detected SCFAs in pigs at 7 days, 14 days, and 14 days after DSS-induced model establishment, demonstrating that sodium isobutyrate can gradually increase the SCFA content in pigs and has a significant effect on colonic metabolism after prefeeding for 14 days. This effect is closely related to the potential increase in the abundance of probiotics, including *Lactobacillus, Christensenellaceae_R-7_group*, *B. porcorum,* and *Ruminococcaceae UCG-005*, in the NaIB + DSS group. These probiotics can produce SCFAs and regulate the intestinal barrier, improving intestinal immune function [75]. These findings may explain why the clinical manifestations of pig colon injury are not severe.

SCFAs act as signaling molecules that connect microorganisms to the host immune system [76]. SCFAs are produced by microbial metabolism within the gut, and their concentrations in the lumen correlate with the abundance of beneficial bacteria in the intestines [77]. On the basis of the findings of 16S rRNA and metagenomic analysis, the levels of the core gut strain *L. reuteri* were found to be elevated. Additionally, we conducted a metabolomic analysis of the colonic contents to identify functional microbial metabolites. Our study revealed that prefeeding with isobutyrate resulted in a significant separation of microbial metabolite levels in both the normal and DSS-induced colitis groups. To more rigorously screen for differentially abundant metabolites, we identified significantly different metabolites in both the normal and DSS-induced colitis groups, ultimately identifying 18 differentially abundant metabolites that exhibited significant changes between these groups. By combining metagenomics with VIP values, we focused on 2 significantly differentially abundant metabolites, namely, 3-HB and ILA. 3-HB is produced by the decomposition of SCFAs and has been shown to play important roles in delaying aging, improving osteoporosis, and alleviating UC [78,79]. Isobutyrate can alter the composition of the host gut microbiota, promote the production of various SCFAs, metabolize into the blood, and participate in circulation, thereby allowing the body to produce more 3-HB. Indole derivatives are significant products of the gut microbiota and have the potential to effectively improve colitis [80,81]. They are mostly produced via the metabolism of tryptophan by *Lactobacillus*, which has beneficial effects on the host [82]. For example, *Lactobacillus* and its metabolite ILA can induce the differentiation of CD4^+^ T cells and regulate host immune function directly or indirectly [83]. We observed a significant increase in ILA levels after isobutyrate prefeeding, and the correlation analysis of differentially abundant metabolites and the core microbiota indicated a highly positive correlation between *L. reuteri* and ILA levels. *L. reuteri* has been identified as an immunomodulatory probiotic that can mediate the production of immune factors and promote the proliferation of intestinal regulatory T cells [84]. In general, the metabolism of tryptophan by intestinal bacteria is decreased in patients with IBD, leading to disruption of the intestinal microenvironment [85]. In this study, *L. reuteri* was the main strain involved in tryptophan metabolism. Metabolism yields the anti-inflammatory metabolite ILA, which is one of the key factors in alleviating IBD.

To better elucidate the biological functions of the differentially abundant metabolites, KEGG functional enrichment analysis was performed. By integrating the KEGG analysis of the normal and DSS-treated samples, a total of 11 common pathways were identified, including tryptophan metabolism, linoleic acid metabolism, protein digestion and absorption, and retrograde endocannabinoid signaling. *L. reuteri* metabolizes tryptophan to produce ILA, which improves host health; thus, the tryptophan metabolism KEGG pathway was enriched in these samples. In addition, research has shown that linoleic acid, which is an unsaturated fatty acid, is a functional fatty acid that has been indicated to improve the immune function of animals [86]. Moreover, we found through metagenomics and binning analysis that isobutyrate can promote metabolic pathways such as tryptophan and butyrate metabolism in *L. reuteri*. The enrichment of these metabolic pathways indicates that isobutyrate may facilitate microbe-induced physiological adaptations to mitigate inflammation in the host by modulating its own metabolism and metabolite production under conditions of DSS-induced colitis.

Isobutyrate can affect the gene or protein expression of the host by modulating the gut microbiota. Previous studies have shown that microbe-derived indole derivatives exert various effects, such as anti-inflammatory and antitumor effects, and can protect against colitis by activating AhR [85,87]. To confirm this hypothesis, we conducted tests on the AhR pathway and found that AhR was activated in colon tissues. We also assessed the expression levels of genes downstream of AhR (*CYP1A1*, *CYP1A2*, and *CYP1B1*) and found that their expression levels were increased in the colon, indicating activation of the AhR pathway in the colon. Furthermore, we treated IPEC-J2 cells with sodium isobutyrate and found that AhR and its pathway were not activated. Previous studies have shown that SCFAs do not directly activate the AhR receptor. For example, studies indicate that butyrate does not directly interact with AhR but indirectly activates the AhR receptor by promoting the production of AhR ligands (such as indole derivatives) [88,89], which is consistent with our results. We further confirmed these findings in IPEC-J2 cells and found that isobutyrate significantly inhibited the mRNA expression of IDO1. IDO1 can metabolize 99% of dietary tryptophan [90]. Isobutyrate may increase the intestinal tryptophan concentration by limiting the consumption of tryptophan by intestinal epithelial cells, leading to the proliferation of tryptophan-utilizing bacteria such as *Lactobacillus* [37]. This may be the mechanism by which isobutyrate promotes the growth of *L. reuteri* and other *Lactobacillus* strains in interaction with the host. SCFAs have positive effects on the differentiation of host immune cells, immune metabolism, and regulation of susceptibility to other pathogens [91]. Our previous research indicated that BSCFA homeostasis is maintained in Min pigs with strong resistance to colitis, which may promote the expression of CD4^+^ T cells [22]. CD4^+^ T cells can restore immune activity, and the higher the abundance of immune cells is, the higher the level of immune function. In addition, *L. reuteri* can activate host Foxp3^+^ CD4^+^ T cells to promote wound healing [92]. Furthermore, we assessed the abundance of CD4^+^ T cells and CD8^+^ T cells in the colon, and the results revealed a significant increase in the number of CD4^+^ T cells in the NaIB + DSS and NaIB groups. Foxp3^+^ T cells are the main subset of regulatory T cells (Tregs), and Tregs are the main subset of CD4^+^ T cells that inhibit host immunity. We further assessed Foxp3 expression and found that the expression of Foxp3 was significantly increased in the NaIB + DSS and NaIB groups. Therefore, we speculate that isobutyrate may regulate the AhR signaling pathway and modulate the immune function of CD4^+^ T cells by promoting the production of ILA by *L. reuteri*.

In addition to regulating the gut microbiota, isobutyrate can also directly activate GPR109A to improve intestinal barrier function. One of the primary mechanisms by which SCFAs function is promoting the function of the intestinal barrier by activating GPCRs [52,93]. However, the extent to which isobutyrate can activate GPCRs has not been extensively examined. As a result, we conducted tests on 3 key GPCRs and discovered that prefeeding with sodium isobutyrate significantly activated the expression of GPR109A, which may be the primary mechanism of action for sodium isobutyrate. Previous studies have shown that acetate can activate GPR43 and that butyrate and 3-HB are important ligands that activate GPR109A [23,94]. In this study, isobutyrate caused an increase in the abundance of intestinal microbes and their metabolites, with significant increases in butyrate and 3-HB, which may also be important reasons for the activation of GPR109A. To investigate whether isobutyrate can bind to GPR109A independently, we conducted experiments in IPEC-J2 cells. The results demonstrated that the addition of 1 to 4 mM sodium isobutyrate alone significantly activated GPR109A. These findings indicate that isobutyrate has dual effects, as it not only participates in the activation of GPR109A through microbiota interactions but also independently activates GPR109A. Finally, the anti-inflammatory and immune-regulating effects of SCFAs are particularly prominent [29]. Patients with IBD have conditions such as intestinal inflammation and immune dysregulation [95]. IBD is often associated with the activation of Toll-like receptors (TLRs), and inhibition of this signaling pathway can effectively alleviate colitis [96]. We investigated the alleviation of these conditions after prefeeding with isobutyrate. We assessed the expression of inflammatory proteins and found that the addition of isobutyrate decreased the expression of key proteins, such as TLR4/MyD88/NF-κB, as well as the level of the NLRP3 inflammasome. When intestinal barrier function is impaired, LPS can exacerbate intestinal inflammation through proinflammatory signaling pathways [97,98]. Our results indicate that isobutyrate can maintain intestinal barrier function and optimize the microbiota structure to inhibit the production of harmful bacteria, thereby reducing LPS production and subsequently suppressing related proinflammatory pathways (TLR4/MyD88/NF-κB) [99,100]. Overexpression of IL-1β and TNF-α can lead to immune dysfunction in the body, resulting in host inflammation. The addition of isobutyrate significantly inhibited this phenomenon. Mice that are deficient in IL-10 exhibit spontaneous colitis [101], and our experiments revealed that the addition of isobutyrate increased host serum and colon levels of IL-10. On the basis of the decreased levels of isobutyrate in patients with IBD, colitis mice, and pigs, we speculate that supplementation with isobutyrate may be an effective way to alleviate colitis. Surprisingly, verification experiments indicated that isobutyrate can confer resistance to colitis in pigs through host–microbiota interactions. This study provides theoretical support for research on BSCFA-isobutyrate and proposes a new treatment method for alleviating IBD in clinical patients.

## Methods

### Animals and sample collection

Thirty-two crossbred pigs (42 days of age, castrated male) with similar physiological conditions were sourced from commercial farms and housed individually in stainless steel metabolic cages equipped with feeding troughs and automatic water dispensers. During the formal experimental period, the pigs were randomly assigned to 2 groups based on their body weight: the control group (CON) and the NaIB group. The CON group was fed the basal diet (Table S2), and the NaIB group was fed the basal diet + 1,000 mg/kg NaIB. On day 15, both pigs in the CON and NaIB groups were randomized into 2 treatment subgroups: sterile water and DSS (4% DSS in 100 ml of sterile water) groups. In these subgroups, the treatments were administered via tube feeding for 5 days, with the measurements doubled on the first day. The groups that were treated with DSS were considered the DSS and NaIB + DSS groups. The dose of DSS was chosen according to Bassaganya-Riera and Hontecillas [102]. Pig feces were collected on days 7 and 14 of the formal experiment and used to determine changes in SCFA levels in the feces. On day 20 (65 days of age), all the pigs were euthanized. Tissue samples from each pig were collected immediately after slaughter, and blood samples were drawn from the anterior vena cava. The blood samples were left to stand for 10 min before being centrifuged at 3,000 *g* for 10 min. The resulting supernatant (serum) was collected and stored at −80 °C. Tissue samples were collected from the distal colon (10 cm proximal to the anus). The colonic digesta was collected, the colon was washed with physiological saline, and the colonic mucosa was quickly scraped on ice. All collected samples were immediately placed in cryovials and stored at −80 °C for further analysis. Additionally, a 2-cm circular segment of the washed distal colon was fixed in 4% paraformaldehyde for further examination, and 3 mm × 1 mm pieces were sectioned and placed in 2.5% glutaraldehyde for additional testing. Similarly, proximal colon samples (10 cm distal to the cecum) and mid-colon samples were preserved in 4% paraformaldehyde for subsequent analysis.

### Disease activity index

The DAI score was calculated using the formula: (body weight loss score + stool consistency score + hematochezia score)/3. The scoring criteria are detailed in Table S3. Fecal occult blood detection was performed via the pyramidon semiquantitative method.

### Organ index

The spleen and colon were excised, weighed, and used to calculate the organ index according to the following formula:

$$\begin{array}{c} \text{Organ index}\;\left(\%\right)=\frac{\text{organ weight}}{\text{body weight}}\times 100\% \end{array}$$ (1)

### Histological analysis

The colon tissue is first fixed in 4% paraformaldehyde solution for 24 h, followed by dehydration through a graded ethanol series. The tissue is then clarified with xylene and subsequently embedded in molten paraffin at 60 °C. After the tissue is solidified, it is sectioned into 4- to 5-μm-thick slices. The sections are then deparaffinized by immersing them in xylene to remove the paraffin, followed by rehydration through a graded ethanol series. The tissue sections are stained with hematoxylin solution for approximately 5 to 10 min, rinsed with running water to remove excess hematoxylin, and then stained with eosin solution for about 2 to 5 min. After rinsing with water, the sections are dehydrated, clarified with xylene, and mounted with a coverslip for subsequent observation. The histopathological scoring criteria were adapted from previous studies [103,104], as detailed in Table S4. Briefly, histopathological scoring of colonic tissues was performed by assessing epithelial damage/erosion, damage of crypts, distortion of crypts, connective tissue hyperplasia, infiltration of inflammatory cells, and edema in the submucosa. Each criterion was graded on a scale from 0 to 3 (0 = none; 1 = mild; 2 = moderate; 3 = severe). To identify goblet cells in the colonic epithelium, the colonic tissues sections were stained with Alcian blue according to the manufacturer’s instructions (Servicebio, Wuhan, China). The slides were stained with Alcian blue solution for 10 to 15 min, washed with distilled water, and dehydrated with anhydrous ethanol and xylene. An imaging system (Winmedic, Shandong, China) was used to capture photomicrographs of the tissue sections under 40/200× magnification. Counting of goblet cells was performed in 10 randomly selected intact crypts per pig.

### TUNEL assay

TUNEL staining of colon sections was performed with a kit (Roche Molecular Systems, MA, Switzerland) according to the instructions, cell nucleus staining was performed, and a fluorescence microscope (Olympus, Tokyo, Japan; 100×) was used to capture the images.

### TEM analysis

Colon tissues were fixed in 2.5% glutaraldehyde for 4 h, followed by washing with phosphate-buffered saline (PBS). The tissues were then further fixed in PBS supplemented with 1% osmium tetroxide for 2 h at room temperature, followed by washing with PBS. The tissues were subsequently dehydrated through a graded ethanol series, embedded overnight in pure epoxy resin, and then placed in fresh epoxy resin. Finally, the samples were cured at 60 °C for 48 h in an oven to complete the embedding process. Sections measuring 80 nm in thickness were cut via a diamond knife on an ultramicrotome. Images were captured via a transmission HT7700 electron microscope manufactured by Hitachi in Tokyo, Japan. The colonic brush border length was averaged after the length of the brush border was measured in 3 randomly selected fields of view for each pig.

### Microbiota data analysis

The 16S rDNA analysis method is consistent with those of previous studies, and detailed methods can be found in the Supplementary Materials [105]. Alpha and beta diversity metrics were calculated on the basis of standardized sequence counts. The feature abundance was normalized to relative abundance using the SILVA classifier. Genes were sequenced on the ImageGP 2 platform [106], and diversity indices were estimated with EasyAmplicon via the R package v4.3 [107,108].

### Metagenomic analysis

The methods used for DNA extraction and sequencing were the same as those used in previous studies; see the Supplementary Materials for details. The Wilcoxon test was used to identify the differentially abundant species, and significant differences were indicated by *P* < 0.05 and a log_2_-fold change > 1. The assignment of microbial functions was performed by using the KEGG database. Metagenomic binning was performed via the MetaWRAP pipeline; for the test methods, refer to previous studies [109].

### Quantification of SCFAs

Gas chromatography was employed to measure the SCFA concentrations in a 2-g sample of colonic digesta. Subsequently, 2 ml of deionized water was added, followed by sonication for 5 min and thorough shaking. After 48 h of extraction, the mixture was centrifuged at a speed of 10,000 *g* per minute for 10 min at a temperature of 4 °C. The resulting supernatant was then filtered 3 times through a 0.22-μm membrane. To this filtered solution, a mixture of metaphosphoric acid and crotonic acid was added at a ratio of 5:1, and the mixture was thoroughly mixed. The solution was once again subjected to centrifugation and filtration and finally stored at 4 °C. The resulting product was injected into a high-performance gas chromatograph for analysis via a capillary column of HP-INNOwax (Catalog No. 19091N-213).

### Colonic metabolite analysis

The method for determining colon metabolites was consistent with that used in previous studies [22]; please refer to the Supplementary Materials for details. The differentially abundant metabolites simultaneously satisfied the following criteria: Fold change ≥ 1.5 or ≤ 1/1.5, *P* value < 0.05, and VIP ≥ 1.

### Gene expression analysis by RT-qPCR

Total RNA was isolated using TRIzol reagent (Takara, Japan) and converted to cDNA with the PrimeScript RT kit (Takara, Japan). RT-qPCR was performed on an Applied Biosystems 7500 Real-Time PCR platform with SYBR Green Mix (Takara, Japan), using β-actin as an internal control. The PCR system and reaction conditions are detailed in Table S5. Relative gene expression levels of *AhR*, *CYP1A1*, *CYP1A2*, *CYP1B1*, *IDO1*, *IDO2*, *TDO*, *GPR41*, *GPR43*, *GPR109A*, *ZO-1*, *Occludin*, and *Claudin-1* were calculated via the 2^–ΔΔCt^ method [110]. The primers used are shown in Table S6.

### Enzyme-linked immunosorbent assay

We measured the protein expression of D-LA, DAO, LPS, and cytokines (IL-1β, IL-10, and TNF-α) in colonic homogenates using enzyme-linked immunosorbent assay kits (Hnybio, Shanghai, China), adhering strictly to the protocol provided in the manual.

### Immunohistochemistry and immunofluorescence

The procedures were performed according to a previously described protocol [29]. Briefly, the colon tissue is first fixed in 4% paraformaldehyde solution for 24 h, followed by dehydration through a graded ethanol series. The tissue is then clarified with xylene and subsequently embedded in molten paraffin at 60 °C. After the tissue is solidified, it is sectioned into 4- to 5-μm-thick slices. The sections are then deparaffinized by immersing them in xylene to remove the paraffin, followed by rehydration through a graded ethanol series. Antigen retrieval was performed by the heat-induced epitope retrieval method. The sections were treated with primary antibodies and incubated overnight at 4 °C. The following antibodies were used for immunohistochemistry: anti-CD4 (Abcam, ab133616, 1:200), anti-CD8b (Abcam, ab228965, 1:250), and anti-Foxp3 (Wanleibio, WL00721, 1:200). The following antibodies were used for immunofluorescence: anti-Claudin-1 (ABclonal, A11530, 1:100), MUC-2 (Servicebio, GB120002-100, 1:500), and Ki67 (Servicebio, GB121141-100, 1:500). For immunohistochemistry, secondary antibodies were conjugated to horseradish peroxidase; for immunofluorescence, fluorescently labeled secondary antibodies were used. Images were obtained with a digital slide scanner (immunohistochemistry, 100×) or a Leica SP5 confocal microscope (immunofluorescence, 200×). Immunohistochemical image analysis was performed via ImageJ software (ImageJ 1.8.0, NIH, USA). The results are expressed as the average optical density (AOD), calculated as follows: AOD = integrated optical density/area of target protein distribution [111].

### Western blot analysis

Proteins were extracted from pig colonic tissue using radioimmunoprecipitation assay lysis buffer. The protein concentration was measured using a bicinchoninic acid protein assay kit. After sodium dodecyl sulfate–polyacrylamide gel electrophoresis, the gel strips were sectioned by molecular weight and transferred to polyvinylidene difluoride membranes. The membranes were then blocked in TBST (Tris-borate-sodium Tween-20) containing skim milk powder at 35 °C for 2 h. Then, primary antibodies were applied at 4 °C for 12 h. Following TBST washes, the membrane was treated with the secondary antibody for 2 h and then subjected to additional TBST washes. Protein visualization was performed with the BeyoECL Star Fluorescence Detection Kit and UVItec gel imaging system. The above reagents were obtained from Beyotime Biotechnology (Shanghai, China). Protein intensity was quantified via ImageJ software. The types, manufacturers, and dilution ratios of the antibodies used are shown in Table S7.

### Cell culture, cytotoxicity assessment, and transfection protocols

IPEC-J2 cells were cultured in Dulbecco’s modified Eagle’s medium/F12 supplemented with 8% fetal bovine serum, 1 μg/ml epidermal growth factor, 1% insulin-transferrin-selenium, 100 μg/ml streptomycin, and 100 IU/ml penicillin at 37 °C in a 5% CO₂ incubator, and passaged or cryopreserved upon reaching ~80% confluence. To determine the cytotoxicity of isobutyrate, a 32 mM stock solution was diluted to 1 mM, and cells (5×10^3^ per well) were seeded in 96-well plates and cultured overnight. Following incubation with serial dilutions of test compounds, 10 μl of CCK-8 solution was added for 2 h, and the absorbance at 450 nm was measured to calculate the maximum nontoxic concentration. On the basis of the findings, sodium isobutyrate (1 to 4 mM) and ILA (applied at previously established safe concentrations) were selected for subsequent cell treatments [112]. For transfection, cells (1×10^5^ per well) seeded in 24-well plates at ~60% confluence were transfected with Lipofectamine 8000 using siRNA or plasmids, following the manufacturer’s instructions. After 6 h, the medium was replaced with complete medium, and the cells were subjected to further treatments for the indicated durations.

### Statistical analysis

Data analysis primarily involved one-way analysis of variance with Tukey’s test via GraphPad Prism 10.2 (GraphPad Software Inc., USA). Student’s unpaired *t* test was used for 2-group dataset analysis. The data are expressed as the means ± SEMs, with the threshold for statistical significance set at an adjusted *P* value < 0.05. 0.05 < *P* < 0.1 was considered to indicate a significant trend. The different letters represent significant differences (*P* < 0.05). **#**0.05 ≤ *P* < 0.1, *P < 0.05, **P < 0.01, ***P < 0.001.

## Ethical Approval

All experimental procedures involving animals in this study were approved by the Institutional Animal Care and Use Committee of Northeast Agricultural University (approval number: NEAU-2013-9).

## Data Availability

Detailed data are provided in the main text and in the Supplementary Materials. The additional data used to support the findings of this study are available from the corresponding authors upon request.
