# Supplementary material for: Isobutyrate Confers Resistance to Inflammatory Bowel Disease through Host–Microbiota Interactions in Pigs
Source: Research (Wash D C). 2025 May 8;8:0673. doi: 10.34133/research.0673 (PMC12059313; doi:10.34133/research.0673)
Supplement: Supplementary 1 — Supplementary Text Figs. S1 to S8 Tables S1 to S7 [file research.0673.f1.docx]

**Isobutyrate confers resistance to inflammatory bowel disease through host-microbiota interactions in pigs**

Example: Xiuyu Fang et al.

*Corresponding author. Email: shibaoming1974@163.com; tengteng@neau.edu.cn

**This file includes:**

Supplementary Text

Figures S1 to S8

Tables S1 to S7

**Supplementary Text**

***DNA extraction***

Microbial genomic DNA was extracted from each faecal sample via a DNA isolation kit (D4015, Omega, Inc., USA) following the manufacturer's instructions. The DNA was eluted with 50 µL of elution buffer and stored at -80 °C until PCR was performed by LC Biotechnology Co., Ltd. (Hangzhou, China).

***PCR amplification and 16S rRNA sequencing***

PCR amplification targeting the V3‒V4 region of 16S rRNA was performed with the primers 341F and 805R. After amplification, 2% agarose gel electrophoresis was performed to verify the fragment size. Adapter sequences and barcodes were added for library construction, followed by quality checks with an Agilent 2100 Bioanalyzer and an Illumina library quantification kit. Libraries with concentrations above 2 nM were sequenced on an Illumina NovaSeq 6000 sequencer in PE250 mode. Paired-end reads were allocated to samples according to unique barcodes and then concatenated with FLASH after barcode and primer removal. Quality control was performed via fqtrim (v0.94), and chimeric sequences were removed via Vsearch (v2.3.4). The feature table and sequences were generated with DADA2 (2019.7).

***Construction of a mouse model of colitis***

I. A total of 24 female BALB/c mice (Liaoning Changsheng Biotechnology Co., Ltd.) aged 6-8 weeks were randomly divided into the following three groups, with 12 mice in each group: (1) the control group (CON) and (2) the DSS treatment group (DSS).

The first phase consisted of a 7-day pretreatment period. The mice were fed base diets during the trial. The second phase lasted for five days and started on day 8. During this period, the CON group consumed drinking water ad libitum, while the DSS group consumed a 4% DSS solution ad libitum.

The mice had free access to tap water and standard commercial mouse feed. Mouse body weight and faecal morphology were monitored daily. On the morning of the 13^th^ day, the mice were humanely euthanized via CO_2_ inhalation. The abdominal cavity was then promptly opened to separate the colon and the containing digesta samples. The colon and digesta samples were subsequently flash-frozen in liquid nitrogen and stored at -80 °C for future analysis.

***Metagenomic analysis***

Total DNA was extracted from the samples by using the Fecal Genome DNA Extraction Kit (AU46111-96BioTeke, China). DNA libraries were constructed by using the TruSeg Nano DNA Library Preparation Kit-Set (#FC-121-4001, Illumina, USA) following the manufacturer's instructions. The metagenome libraries were then sequenced on an Illumina NovaSeg 6000 platform with PE150 at LC-Bio Technology Co., Ltd. (Hangzhou, China). Sequencing adapters were removed from demultiplexed raw sequences via Cutadapt (v1.9). The low-quality reads (quality scores<20), short reads (<100 bp), and reads containing more than 5% “N” bases were subsequently trimmed via the sliding window algorithm method in fqtrim (v 0.94). The quality-filtered reads were first aligned to the pig colonic digesta genome by using Bowtie (v2.2) to filter out host contamination. The remaining reads were subsequently subjected to de novo assembly for each sample via MEGAHIT (v1.2.9) and used to annotate microbial functions and taxonomic classification. MetaGeneMark (v3.26) was used to predict the coding sequences (CDSs) of the assembled contigs, and the CDSs of all the samples were clustered via CD-HIT (v4.6.1) to obtain unigenes. DIAMOND (V0.9.14) was used to perform a taxonomic assessment of the microbiota on the basis of the NR database. The Wilcoxon test was used to identify the differentially abundant species, and significant differences were indicated by *P* < 0.05 and a log_2_-fold change > 1. The annotation of microbial functions was performed via the Kyoto Encyclopedia of Genes and Genomes (KEGG).

***Colonic metabolite measurements and bioinformatics analysis***

The harvested colon contents were thawed on ice, and metabolites were extracted with 50% methanol buffer. All the samples were then analysed via LC‒MS according to the instructions for the system, and the metabolites that were eluted from the column were detected via a TripleTOF 5600 Plus high-resolution tandem mass spectrometer (Sciex, Warrington, UK). The online KEGG database annotates metabolites by matching the exact molecular weight data (m/z) of the samples to the molecular weight data in the database. Metabolite identification was verified by using an in-house metabolite fragment profiling library. In this study, we used univariate analysis of fold differences (fold changes) and t tests with BH correction to determine the p value, which was combined with the variable importance for projection (VIP) value obtained via multivariate statistical analysis (PLS-DA), to identify differentially abundant metabolites. The differentially abundant metabolites simultaneously satisfied the following criteria: fold change ≥ 1.5 or ≤ 1/1.5, *P* value < 0.05 and VIP ≥ 1.

***Cultivation of Lactobacillus reuteri***

This strain was isolated by the team members from the intestines of pigs and was cultivated in conventional MRS medium. Co-culture of isobutyrate and *L.reuteri*: The bacterial suspension was adjusted to a concentration of 10^5^ CFU, and different levels of isobutyrate (0-4 mM) were added to the MRS medium. After co-cultivation for 48 hours, the absorbance was measured using a microplate reader. Co-culture of 1% tryptophan and *L.reuteri*: The bacterial suspension was adjusted to a concentration of 10^5^ CFU. The control group does not add tryptophan, while the treatment group adds 1% tryptophan to MRS liquid medium and co-cultures for 48 hours, after which the optical density is measured using a microplate reader.

***Determination of indole-3-lactic acid (ILA)***

The content of ILA in the supernatant of the bacterial solution was determined using high-performance liquid chromatography. Appropriate methods were used for sample pretreatment. The liquid chromatography conditions were as follows: flow rate of the mobile phase: 1 mL/min, column temperature: 35°C, detection wavelength: 280 nm. A standard curve was plotted based on the peak areas of the standard samples, and the peak area of the test samples was further substituted to calculate the concentration of ILA in the supernatant.

***Cell culture***

IPECJ2 cells were cultured in complete DMEM/F12 supplemented with 8% FBS, 1 µg/mL epidermal growth factor (EGF), 1% insulin-transferrin-selenium (ITS), 100 µg/mL streptomycin, and 100 IU/mL penicillin. The cells were cultured at 37 °C in a 5% CO_2_ cell culture incubator and passaged or cryopreserved when they reached approximately 80% confluency after growth.

***CCK-8 method for detecting the cytotoxicity of drugs***

A 32 mM isobutyrate stock solution was prepared and diluted to 1 mM to detect the cytotoxicity of isobutyrate. The cells were seeded at 5×10^3^ per well in a 96-well plate and cultured overnight at 37 °C in a 5% CO_2_ incubator. The next day, a series of diluted test compounds was added, and after the specified incubation time, 10 μL of CCK-8 solution was added to each well and incubated at 37 °C for 2 hours. The absorbance value at 450 nm was measured via an ELISA reader, and the maximum nontoxic concentration of the drug was calculated.

***Cell processing***

We determined the safe concentration range of sodium isobutyrate (1–4 mM) for subsequent experiments. Indole-3-lactic acid was tested at appropriate concentrations on the basis of previous studies to verify its safety for cell treatment (*3*).

***Cell transfection assay***

The cells were seeded at a density of 1×10^5^ cells/well in a 24-well cell culture plate. After overnight incubation, when the cells reached approximately 60% confluency, the details of the cell transfection experiment were as follows: Preparation of the transfection reagent was performed as follows: Two sterile 1.5 mL Eppendorf tubes we labelled as Tube A and Tube B, and 25 µL of serum-free OPTI-MEM medium was added to each tube. Two microlitres of Lipofectamine 8000 were added to Tube A, the bottom of the Tube was gently tapped to mix, and the mixture was allowed to stand at room temperature for 5 minutes. Moreover, an appropriate amount of siRNA molecules or plasmids was added to Tube B, and the mixture was mixed well. The mixture was transferred from Tube B to Tube A, gently mixed 3–5 times with a pipette and allowed to stand at room temperature for 20 minutes. During this time, the cell culture medium was discarded, the cells were washed with PBS twice, the PBS was discarded, and 400 µL of serum-free OPTI-MEM was added to each well. Then, 100 µL of the A/B mixture was added to the cells to be transfected, the mixture was shaken gently to mix well, and the mixture was cultured in an incubator. After transfection for 6 h, the culture medium was discarded, 500 µL of cell culture medium was added to each well, and the samples were subjected to different treatments. The samples were cultured for the specified times.

Primer sequences for functional verification

| Names | Sense (5'-3') | Anti-sense (5'-3') |
| --- | --- | --- |
| NC siRNA | UUCUCCGAACGUGUCACGUTT | ACGUGACACGUUCGGAGAATT |
| GPR109A siRNA | CGAUGUUAAUCAAGAAGCATT | UGCUUCUUGAUUAACAUCGTT |

1. X. Zhao, L. Jiang, X. Fang, Z. Guo, X. Wang, B. Shi, Q. Meng, Host-microbiota interaction mediated resistance to inflammatory bowel disease in pigs. Microbiome 10, 115 (2022).

2. Wen, L. Yang, Z. Wang, X. Liu, M. Gao, Y. Zhang, J. Wang, P. He, Blocked conversion of Lactobacillus johnsonii derived acetate to butyrate mediates copper-induced epithelial barrier damage in a pig model. Microbiome 11, 218 (2023).

3. Zhong S, Sun Y-Q, Huo J-X, Xu W-Y, Yang Y-N, Yang J-B, et al. The gut microbiota-aromatic hydrocarbon receptor (AhR) axis mediates the anticolitic effect of polyphenol-rich extracts from Sanghuangporus. iMeta. 2024;3(2):e180.

**
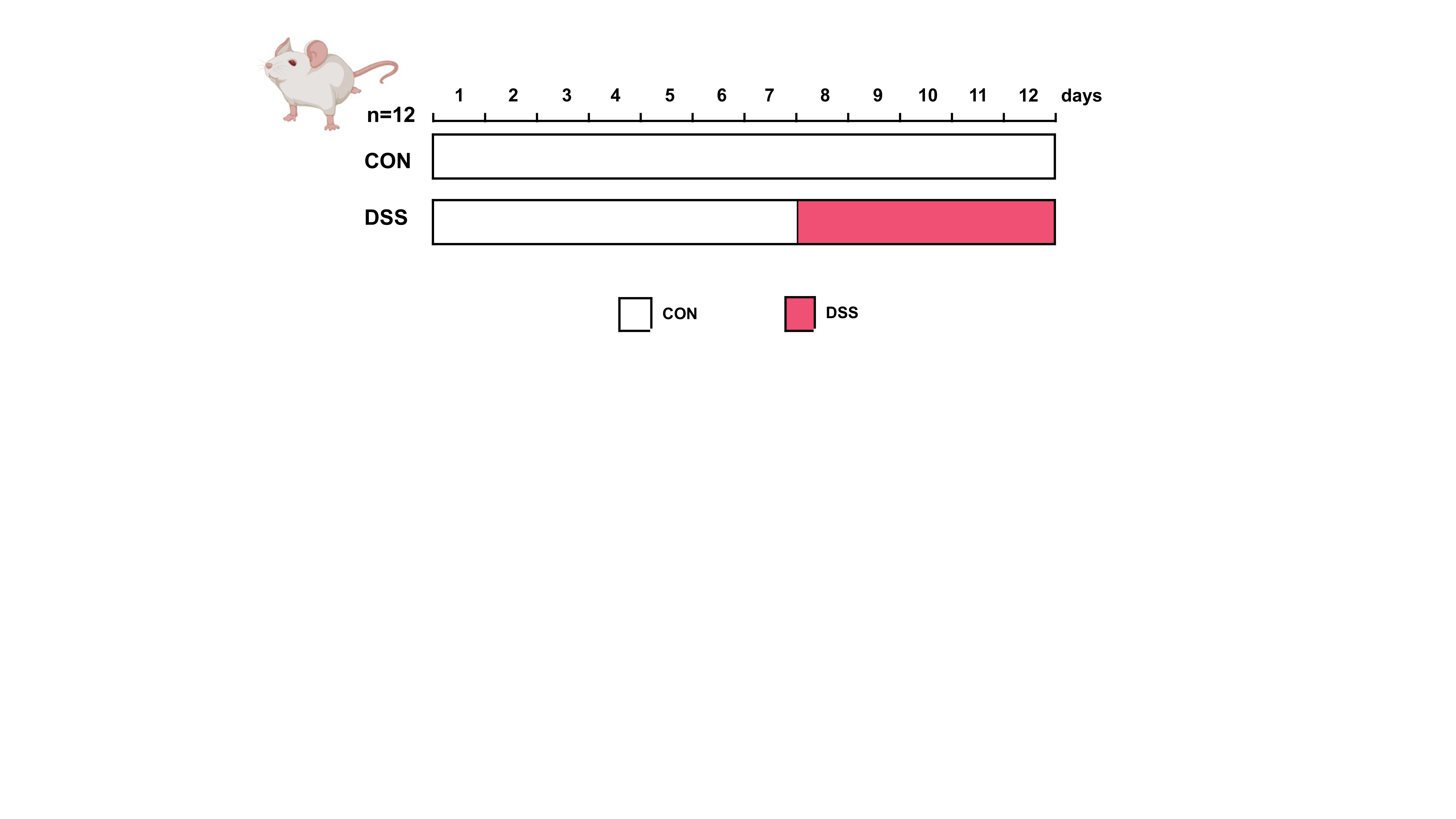
**

**Fig S1.** Flow chart of the DSS-induced colitis mouse test.


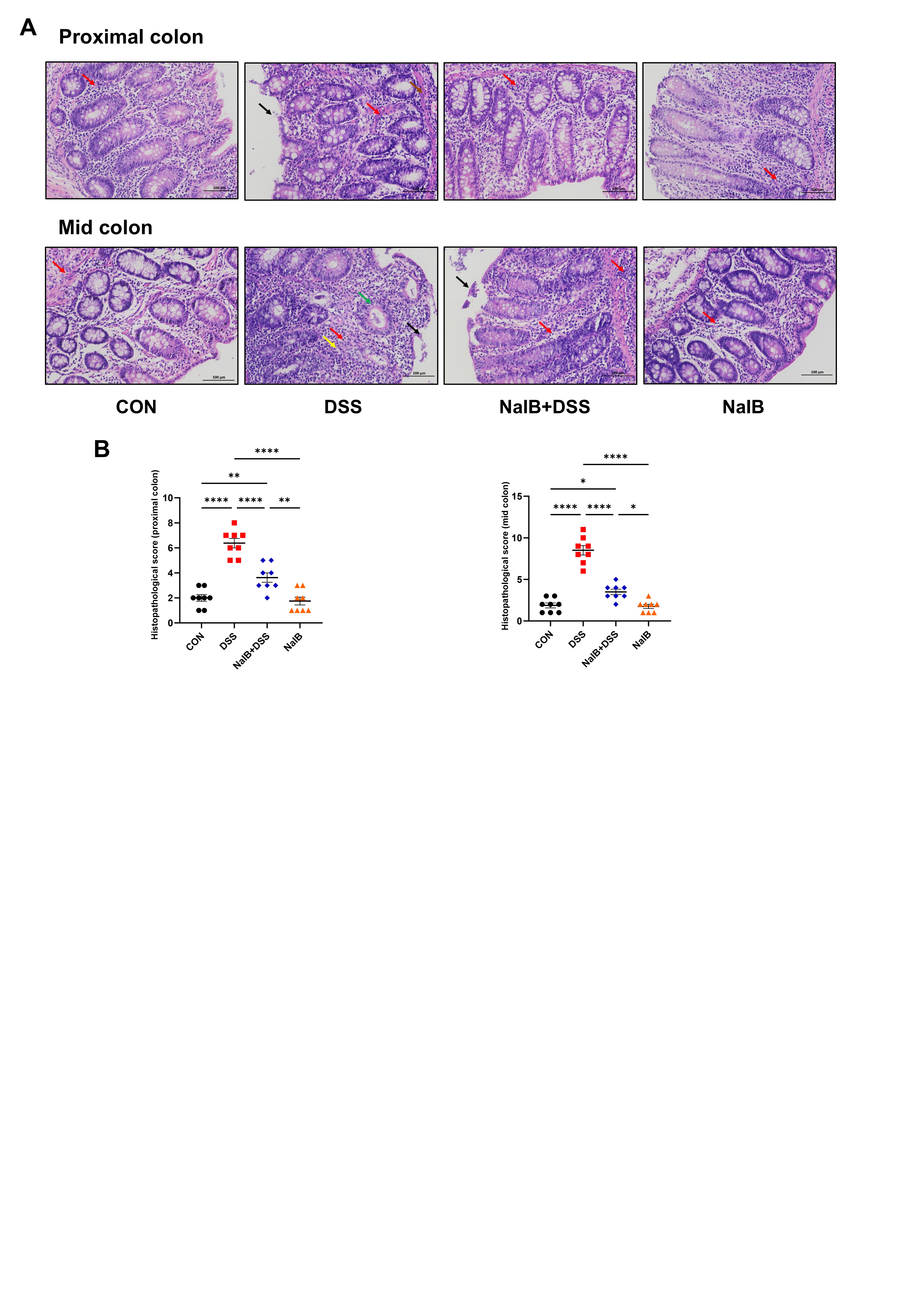


**Fig S2. A** Representative H&E-stained images of the pig proximal colon and mid colon. **B** Histopathological scoring of the pig proximal and mid colon.

Note: Colonic tissue sections were assessed by epithelial damage/erosion (black arrow), damage of crypts (macroscopic observation), distortion of crypts (green arrow), connective tissue hyperplasia (yellow arrow), infiltration of inflammatory cells (red arrow), and edema in the sub-mucosa (brown arrow). A colonic histopathological score was calculated by combining three randomly selected fields of view for each section. The maximum score that could result from this scoring was 18.

**
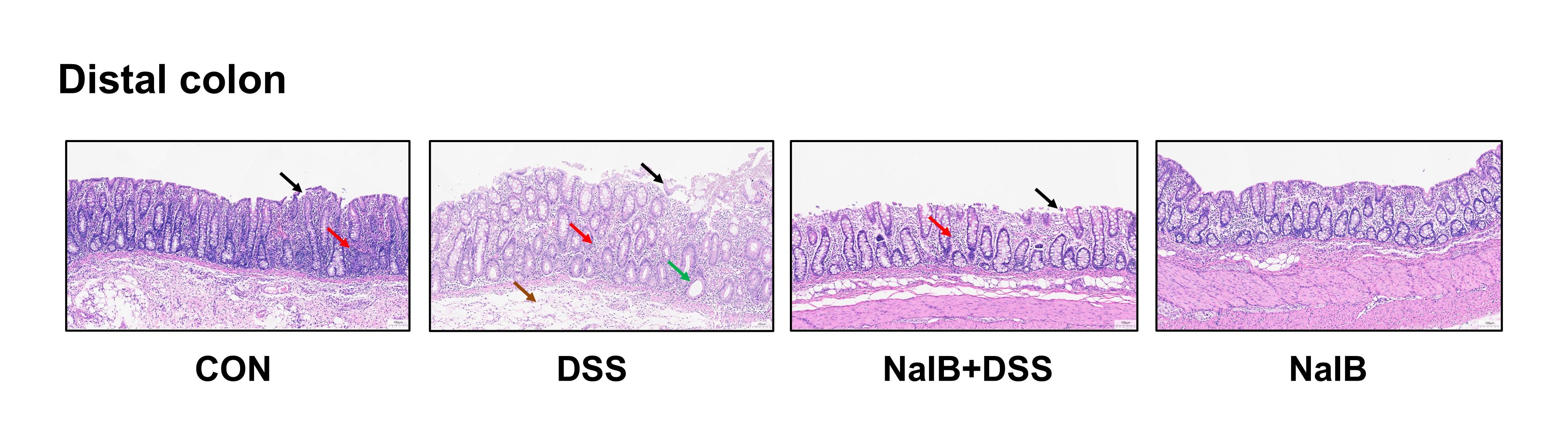
**

**Fig S3.** Representative H&E-stained images of the pig distal colon.

Note: Colonic tissue sections were assessed by epithelial damage/erosion (black arrow), damage of crypts (macroscopic observation), distortion of crypts (green arrow), connective tissue hyperplasia (yellow arrow), infiltration of inflammatory cells (red arrow), and edema in the sub-mucosa (brown arrow). A colonic histopathological score was calculated by combining three randomly selected fields of view for each section. The maximum score that could result from this scoring was 18.

**
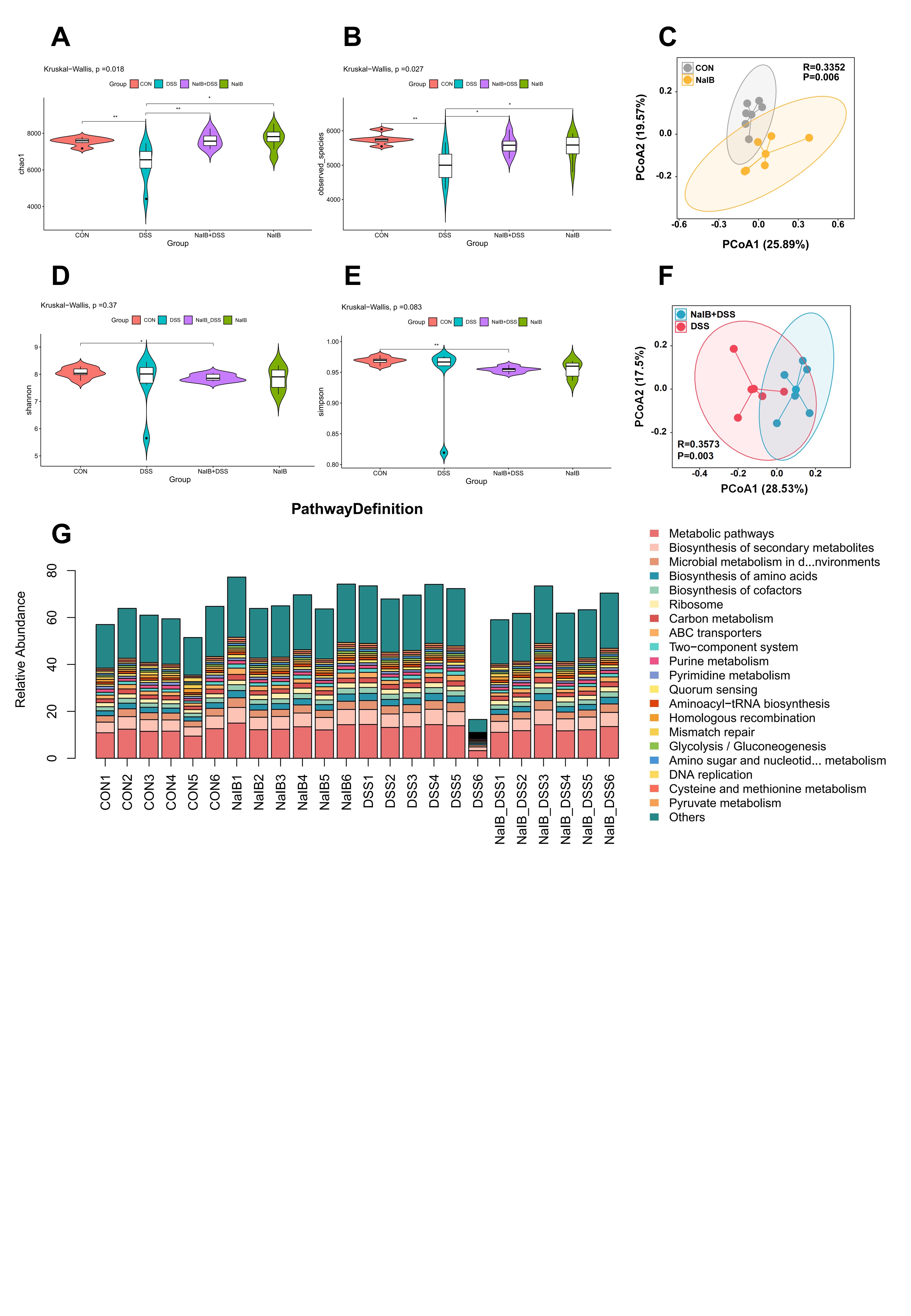
**

**Fig S4. A, B, D and E** Comparison of the Chao1, Observed_Species, Shannon and Simpson indices of the gut microbiota in the CON, DSS, NaIB+DSS and NaIB groups. **C** and **F** Principal coordinate analysis (PCoA) plot of the microbial compositional profiles in pigs after DSS treatment**.** **G** KEGG pathway enrichment analysis of the CON, DSS, NaIB+DSS and NaIB groups. n = 6.

**
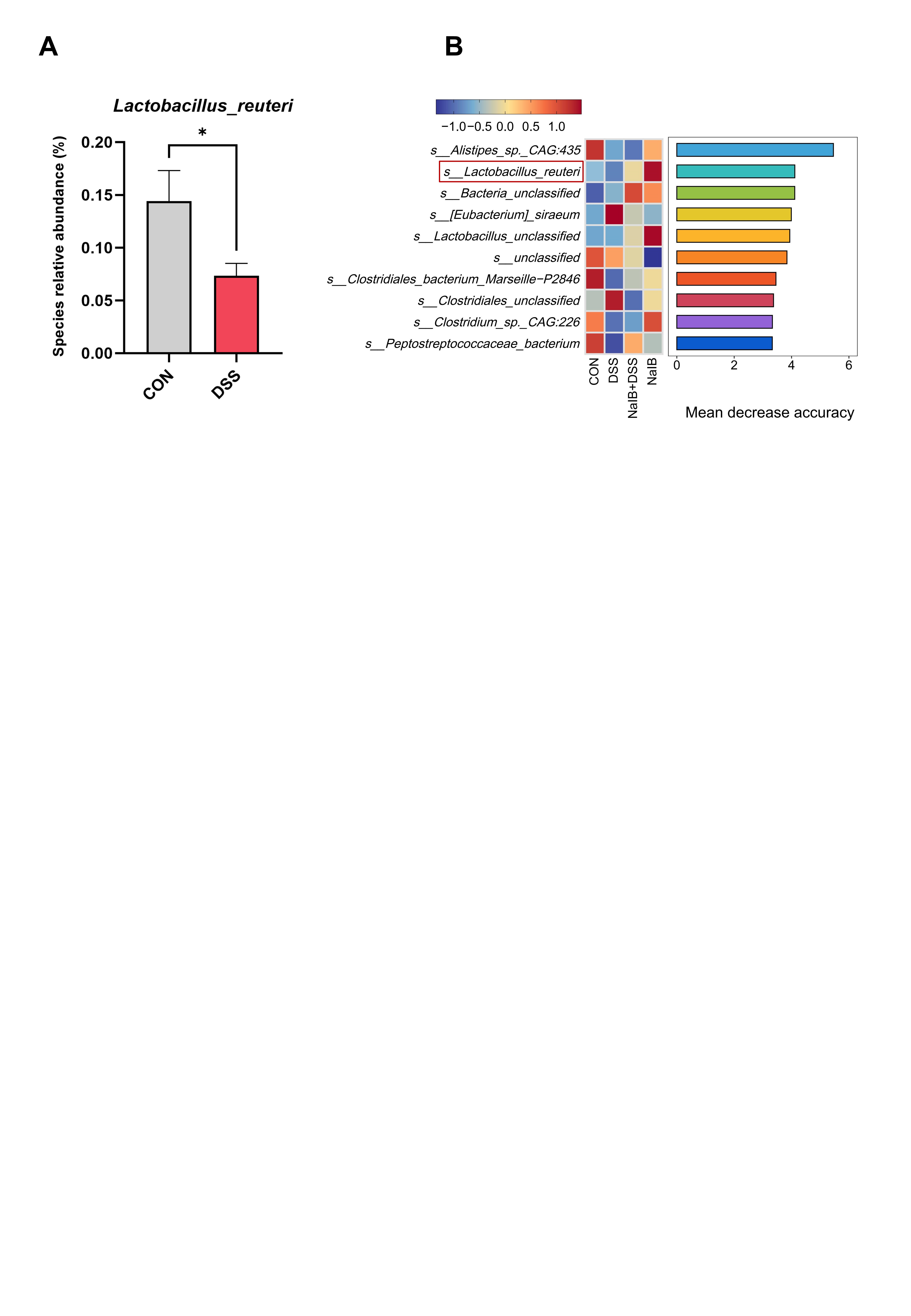
**

**Fig S5. A** The relative abundance of *L.reuteri* in the CON and DSS groups. **B** Random forest analysis of colonic core microbiota. *P* < 0.05 was considered to indicate statistical significance. ** P* < 0.05. n = 6.


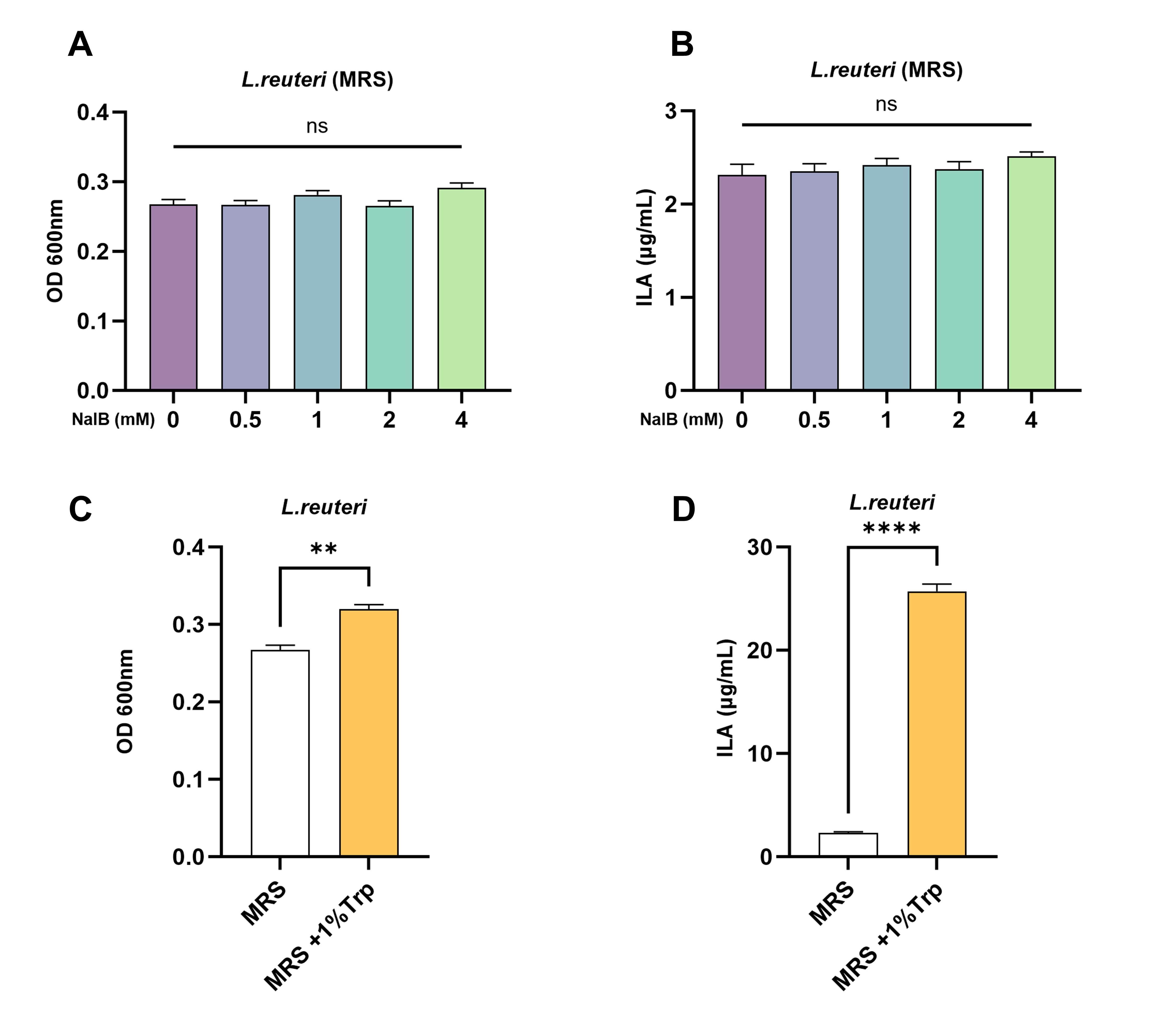


**Fig S6. A** Co-cultivation of different concentrations of isobutyrate with *L. reuteri*. **B** Detection of ILA in the supernatant of bacterial culture after co-culture of different levels of isobutyrate and *L.* *reuteri*. **C** 1% Tryptophan co-cultured with *L. reuteri.* **D** Detection of ILA in the supernatant of bacterial culture after co-culture of 1% tryptophan and *L. reuteri*. *P* < 0.05 was considered to indicate statistical significance. ** P* < 0.05, *** P* < 0.01, *** *P* < 0.001. n = 3.

**
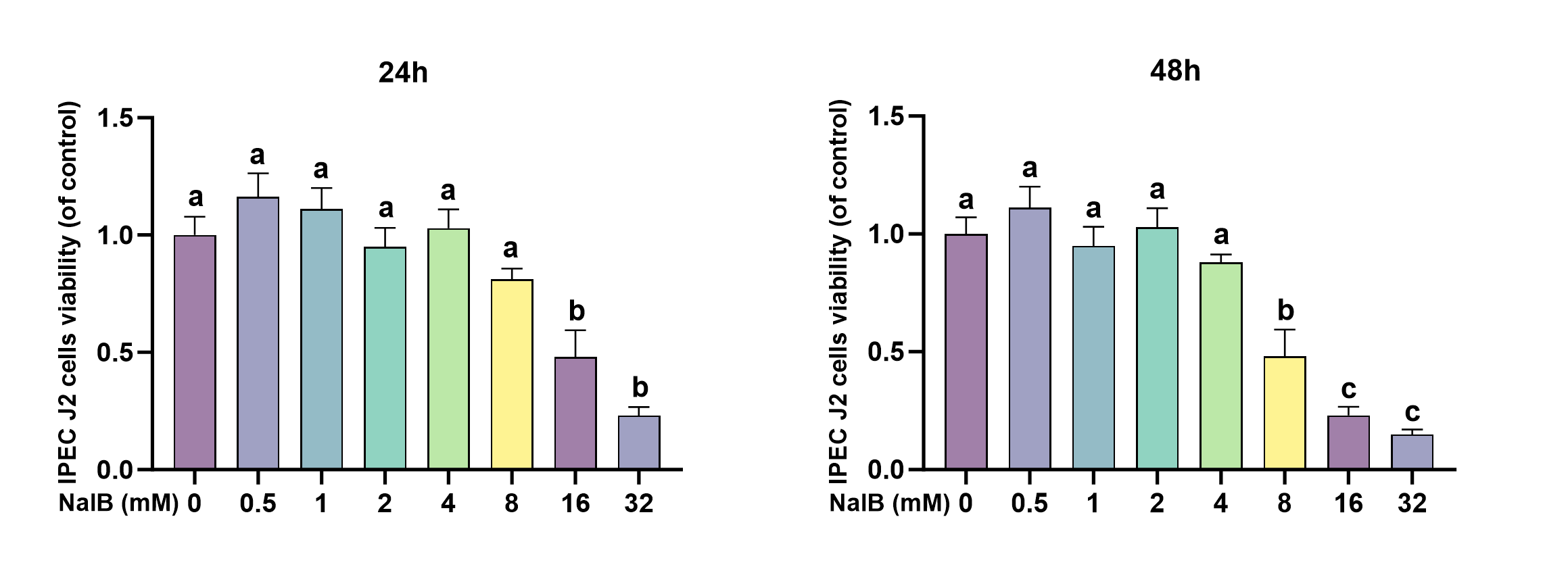
Fig S7.** CCK-8 assays were used to assess the effects of different NaIB concentrations on cell proliferation. The different letters represent significant differences (*P* < 0.05). n = 3.


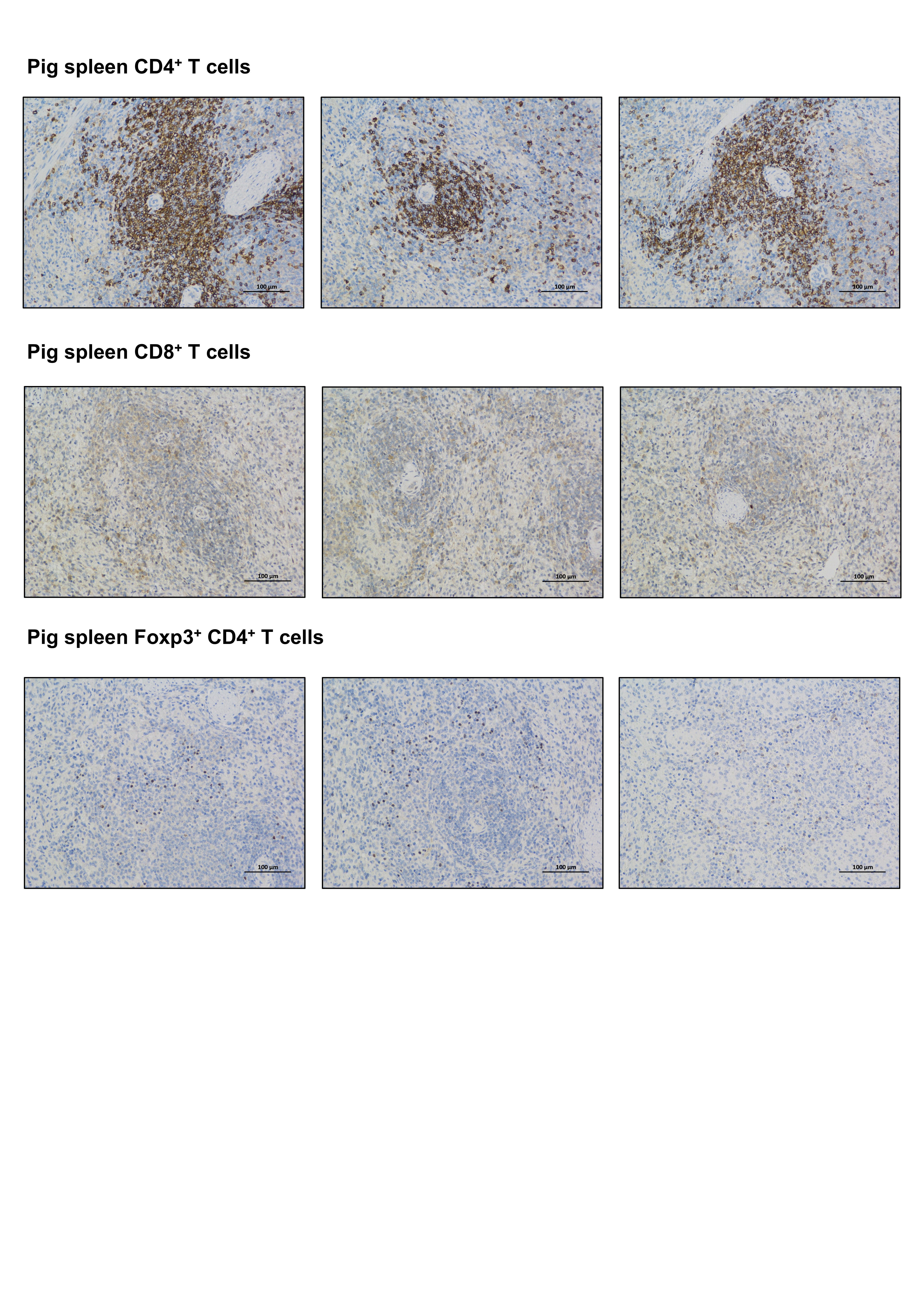


**Fig S8.** CD4^+^ T cells, CD8^+^ T cells, Foxp3^+^ CD4^+^ T cells antibody validation in porcine immune organs (spleen).

**Table S1.** DAI scores.

**
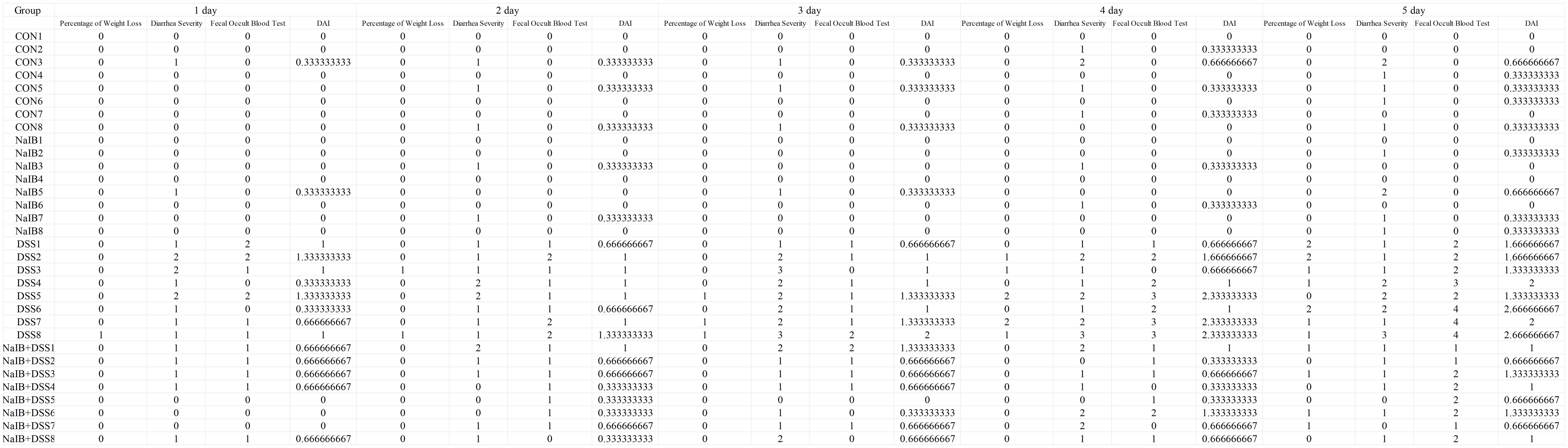
**

**Table S2.** Composition and nutrient levels of the base diet (as-fed basis%).

| Ingredient | Content | Nutrient | Content |
| --- | --- | --- | --- |
| Corn | 45.24 | Net energy (Mcal/kg) | 2.52 |
| Hulled soybean meal | 16.5 | Crude protein | 18.73 |
| Puffed corn | 12.00 | Lysine | 1.25 |
| Rice bran meal | 2.50 | Methionine | 0.37 |
| Whey powder | 5.00 | Threonine | 0.73 |
| Glucose | 2.00 | Tryptophan | 0.22 |
| Whole Puffed Soya | 9.00 | Calcium | 0.74 |
| Fish meal | 3.00 | Total phosphorus | 0.65 |
| Soybean oil | 1.20 | Available phosphorus | 0.37 |
| Calcium dihydrogen phosphate | 0.85 |  |  |
| Limestone | 0.98 |  |  |
| NaCl | 0.4 |  |  |
| L-Lysine HCl | 0.28 |  |  |
| DL-Methionine | 0.05 |  |  |
| Premix | 1.00 |  |  |
| Total | 100.00 |  |  |

Note:^1^ Premix provided the following per kilogram of feed: vitamin A, 12,500 IU; vitamin D_3_, 2,800 IU; vitamin E, 30 mg; vitamin K_3_, 5 mg; vitamin B_1_, 1.5 mg; vitamin B_6_, 3 mg; vitamin B_12_, 40 μg; riboflavin, 15 mg; pantothenic acid, 15 mg; niacin, 40 mg; folic acid, 1 mg; biotin, 0.08 mg; choline chloride, 500 mg; Mn, 4 mg; Fe, 100 mg; Zn, 80 mg; Cu, 6 mg; I, 0.7 mg; Se, 0.48 mg.

^2^ Crude protein values were obtained by analysis, while other values were calculated.

**Table S3.** DAI scoring rules.

DAI scoring rules

| Score | Percentage of Weight Loss | Diarrhoea Severity | Faecal Occult Blood Test |
| --- | --- | --- | --- |
| 0 | 0% (No loss) | Normal stool | No purplish red colour within 2 minutes |
| 1 | 1-5% | Soft stool, formed | Gradual purple-red colouration within 1-2mins |
| 2 | 5-10% | Pasty, unformed | Fuchsia colour within 1min |
| 3 | 10-20% | Liquid, clear separation of faecal water | Fuchsia colour within 10s |
| 4 | >20% |  | Immediate fuchsia colour |

**Table S4.** Histopathological scoring rules.

Histopathological scoring rules

| Lesion  Score | Epithelial damage/  erosion | Damage of crypts | Distortion of crypts | Connective tissue hyperplasia | Infiltration of inflammatory cells | Edema in the sub-mucosa | |
| --- | --- | --- | --- | --- | --- | --- | --- |
| 0 | none | none | none | none | none | none |  |
| 1 | mild | ≤25% | mild | mild | mild | mild |  |
| 2 | moderate | 25%-50% | moderate | moderate | moderate | moderate |  |
| 3 | severe | ≥50% | severe | severe | severe | severe |  |

Note: Colonic tissue sections were assessed by epithelial damage/erosion (black arrow), damage of crypts (macroscopic observation), distortion of crypts (green arrow), connective tissue hyperplasia (yellow arrow), infiltration of inflammatory cells (red arrow), and edema in the sub-mucosa (brown arrow). A colonic histopathological score was calculated by combining three randomly selected fields of view for each section. The maximum score that could result from this scoring was 18.

**Table S5.** PCR system and reaction conditions

PCR System

| PCR Component | Volume |
| --- | --- |
| Phusion Hot Start Flex 2X Master Mix | 12.5 µl |
| Forwards Primer | 2.5 µl |
| Reverse Primer | 2.5 µl |
| Template DNA | 50 ng |
| Add ddH2O to | 25 µl |

PCR Conditions

| Temperature | Time | Number of Cycles |
| --- | --- | --- |
| 98 °C | 30 s |  |
| 98 °C | 10 s | 35 cycles |
| 54 °C | 30 s |  |
| 72 °C | 45 s |  |
| 72 °C | 10 min |  |
| 4 °C | (Hold) |  |

**Table S6.** Primers used for mRNA expression analysis via RT‒qPCR.

| ***Gene names*** | ***Forwards Primer (5’ to 3’)*** | ***Reverse Primer (5’ to 3’)*** |
| --- | --- | --- |
| Pig *AhR* | CCAGTTAGAGCAGCAGCAGCAG | AAAAGGCACGGGTTGGTTAGAGC |
| Pig *CYP1A1* | GGACAAGAGGATGGACGAGAATGC | GGAGATGGCGGTTGTGACTGTG |
| Pig *CYP1A2* | GCTTCACTCTGGTCACTGATGGC | CAGGTAGCAGGAGGAGGAGGAAG |
| Pig *CYP1B1* | CACGATGCGAGCCTTCACCAC | GCACGAGCAGCGATACCAACTC |
| Pig *IDO1* | GGTCTGCTCTATGAAGGCGTTTGG | GGACACCCAGGAGAACATCAAAGC |
| Pig *IDO2* | GCCAGTTCCTCACGGGTTACAAG " | TTGGGCTGCGTCTCTCCTTCC |
| Pig *TDO* | CGAGTGGTGGTGATCCTCAAACTG | ACTGCAAACTCTGGAAGCCTGATG |
| Pig *GPR41* | ACTACTTCTCATCCTCGGGGTT | CTCCACT TCGCTCTTCTTCAGT |
| Pig *GPR43* | TCATGGGTTTCGGCTTCTACAG | GTACTGA ACGATGAACACGACG |
| Pig *GPR109A* | AGCCATCATCTCCTGCCTCCTG | ATCATGCCAGCGGAAGGTATTGC |
| Pig *ZO-1* | TCAAGGTCTGCCGAGACAAC | ATCACAGTGTGGTAAGCGCA  ATCACAGTGTGGTAAGCGCA  ATCACAGTGTGGTAAGCGCA |
| Pig *Occludin* | TTCATTGCTGCATTGGTGAT | ACCATCACACCCAGGATAGC |
| Pig *Claudin-1* | ATGACCCCAGTCAATGCCAG | CAAAGTAGGGCACCTCCCAG |

**Table S7.** Primary antibodies.

| Antibody | Cat. No | Concentration | Supplier |
| --- | --- | --- | --- |
| β-actin | AC026 | WB: 1:10000 | ABclonal, Hubei, China |
| GPR41 | A12636 | WB: 1:1000 | ABclonal, Hubei, China |
| GPR43 | A18592 | WB: 1:1000 | ABclonal, Hubei, China |
| GPR109A | A15611 | WB: 1:1000 | ABclonal, Hubei, China |
| Occludin | A2601 | WB: 1:1000 | ABclonal, Hubei, China |
| Claudin1 | A11530 | WB: 1:1000  IHC-P: 1:100 | ABclonal, Hubei, China |
| ZO-1 | A25306 | WB: 1:1000 | ABclonal, Hubei, China |
| IDO1 | A1614PM | WB: 1:3000 | ABclonal, Hubei, China |
| MUC-2 | GB120002-100 | IHC-P: 1:500 | Servicebio, Hubei, China |
| Ki67 | GB121141-100 | IHC-P: 1:500 | Servicebio, Hubei, China |
| CD4 | ab133616 | IHC-P: 1:200 | Abcam, Cambridge, United Kingdom |
| CD8b | ab228965 | IHC-P: 1:250 | Abcam, Cambridge, United Kingdom |
| Foxp3 | WL00721 | IHC-P: 1:200 | Wanleibio, Liaoning, China |
| AhR | WL02657 | WB: 1:1000 | Wanleibio, Liaoning, China |
| TLR4 | WL00196 | WB: 1:500 | Wanleibio, Liaoning, China |
| MYD88 | WL02494 | WB: 1:1000 | Wanleibio, Liaoning, China |
| NF-κB | WL01273b | WB: 1:1000 | Wanleibio, Liaoning, China |
| p-NF-κB | WL02169 | WB: 1:1000 | Wanleibio, Liaoning, China |
| NLRP3 | WL02635 | WB: 1:1000 | Wanleibio, Liaoning, China |
